# Targeted inhibition of STAT3 induces immunogenic cell death of hepatocellular carcinoma cells via glycolysis

**DOI:** 10.1002/1878-0261.13263

**Published:** 2022-06-27

**Authors:** Ya Li, Zhenwei Song, Qiuju Han, Huajun Zhao, Zhaoyi Pan, Zhengyang Lei, Jian Zhang

**Affiliations:** ^1^ Institute of Immunopharmaceutical Sciences, School of Pharmaceutical Sciences Shandong University Jinan China

**Keywords:** glycolysis, HCC, ICD, immune environment, STAT3

## Abstract

In hepatocellular carcinoma (HCC), the signal transducer and activator of transcription 3 (STAT3) is present in an overactive state that is closely related to tumour development and immune escape. STAT3 inhibition reshapes the tumour immune microenvironment, but the underlying mechanisms have not been fully clarified. We found that STAT3 inhibition could induce immunogenic cell death (ICD) of HCC cells via translocation of the “eat me” molecule calreticulin to the cell surface and a significant reduction in the expression of the “don’t eat me” molecule leucocyte surface antigen CD47. STAT3 inhibition promoted dendritic cell (DC) activation and enhanced the recognition and phagocytosis of HCC cells by macrophages. Furthermore, STAT3 inhibition prevented the expression of key glycolytic enzymes, facilitating the induction of ICD in HCC. Interestingly, STAT3 directly regulated the transcription of *CD47* and solute carrier family 2 member 1 (*SLC2A1*; also known as *GLUT1*). In subcutaneous and orthotopic transplantation mouse tumour models, the STAT3 inhibitor napabucasin prevented tumour growth and induced the expression of calreticulin and the protein disulfide isomerase family A member 3 (PDIA3; also known as ERp57) but suppressed that of CD47 and GLUT1. Meanwhile, the amount of tumour‐infiltrated DCs and macrophages increased, along with the expression of costimulatory molecules. More CD4^+^ and CD8^+^ T cells accumulated in tumour tissues, and CD8^+^ T cells had lower expression of checkpoint molecules such as lymphocyte activation gene 3 protein (LAG‐3) and programmed cell death protein 1 (PD‐1). Significantly, the antitumour immune memory response was induced by treatment targeting STAT3. These findings provide a new mechanism for targeting STAT3‐induced ICD in HCC, and confirms STAT3 as a potential target for the treatment of HCC via reshaping the tumour immune microenvironment.

## Graphical abstract

STAT3 inhibition induces immunogenic cell death (ICD) of HCC cells via translocation of the “eat me” molecule calreticulin to the cell surface and a significant reduction in exposure of the “don’t eat me” molecule CD47. STAT3 inhibition prevented the expression of key glycolytic enzymes, facilitating the induction of ICD in HCC. Significantly, the antitumour immune memory response was induced by treatment targeting STAT3.

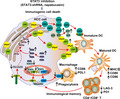

**Abbreviations**
CRT: calreticulin
DC: dendritic cell
ECAR: extracellular acidification rate
eIF2α: eukaryotic translation initiation factor 2α
ER: endoplasmic reticulum
GLUT1: glucose transporter 1
GM‐CSF: granulocyte macrophage‐colony stimulating factor
HCC: hepatocellular carcinoma
HIF‐1α: hypoxia inducible factor‐1 α
HK2: hexokinase 2
HMGB1: high mobility group box 1
HSPs: heat shock proteins
ICD: immunogenic cell death
LAG‐3: lymphocyte activation gene 3 protein
LDHA: lactate dehydrogenase A
‐ODN: decoy‐oligodeoxynucleotide
PBMCs: peripheral blood mononuclear cells
PD‐1: programmed cell death protein 1
PDIA3, also known as ERp57: protein disulfide isomerase family A member 3
PK: pyruvate kinase
PVDF: Polyvinylidene‐Fluoride.
*SLC2A1* also known as *GLUT1*
: solute carrier family 2 member 1
STAT3: signal transducer and activator of transcription 3

## 1 Introduction

Hepatocellular carcinoma (HCC) is the most common form of primary liver cancer and has high morbidity and mortality [1, 2]. In addition, HCC represents the main histological subtype of primary liver cancer, accounting for up to 90% of the total liver cancer burden worldwide [3]. Surgery, chemotherapy, and radiotherapy are widely used for the treatment of HCC, but cannot effectively control recurrence and metastasis. Thus, the ideal therapy for HCC should not only aim at resecting tumour *in situ* safely and effectively, but also removing residual tumour cells by activating the immune system to prevent tumour metastasis and recurrence [2].

Apoptosis can be divided into physiological cell death and immunogenic cell death (ICD) according to the expression of immunogenic molecules on the apoptotic cell surface, which cause immune attack responses [4]. Cells undergoing ICD upregulate the expression of eat me signalling molecules on the cell surface, such as calreticulin (CRT) and protein disulfide isomerase family A member 3 (PDIA3, also known as ERp57). Meanwhile, the heat shock proteins (HSPs), adenosine triphosphate (ATP) and high mobility group box 1 (HMGB1), are also released during ICD induction. All these ICD‐related molecules can stimulate immune cells to recognise and attack tumour cells [5].

Anthracyclines can induce the translocation of calreticulin from the cytoplasm to cell surface in tumour cells, which leads to their recognition by dendritic cells (DCs) and tumour antigen processing and presentation to CD4^+^ T and CD8^+^ T cells, thereby stimulating the antitumour immune response. The translocation of calreticulin requires the cotranslocation of ERp57 [6]. During ICD induced by platinum, anthracycline, or other chemotherapeutic drugs, the degraded tumour‐specific antigens are transported to the cell surface via HSP70 and HSP90, enhancing the presentation of antigens to DCs as well as their interaction with tumour cells [7]. Calreticulin are recognised as “eat me” signals, promoting the phagocytosis of cancer cells by the immune system. By contrast, the glycoprotein CD47 on the tumour cell membrane, which inhibits macrophage‐mediated phagocytosis, is considered a “don't eat me” signal [8]. At the early stage of ICD, numerous molecules of calreticulin and ERp57 translocate to the cell surface, while the expression of CD47 is significantly decreased [9]. The alteration of “don't eat me” and “eat me” signals results in the effective recognition and phagocytosis of tumour cells by DCs and macrophages. Therefore, ICD induction is one of the antitumour strategies that can elicit more effective antitumour immune responses.

The Warburg effect is the metabolic feature of several malignant tumours, which are more prone to glycolysis even in an oxygen‐rich tumour microenvironment. Aerobic glycolysis is the process by which glucose is metabolised by a series of enzymes, such as the solute carrier family 2 member 1 (SLC2A1; also known as GLUT1), hexokinase 2 (HK2), pyruvate kinase (PK), and lactate dehydrogenase A (LDHA), and is also regulated by the hypoxia inducible factor‐1 α (HIF‐1α) [10]. The GLUT1 inhibitor WZB117 can effectively inhibit the proliferation of A549 and MCF7 cell lines *in vitro* [11]. HIF‐1α inhibitors have shown excellent antitumour effects *in vitro* and *in vivo*, either alone or in combination with traditional chemotherapeutic drugs [12, 13]. In addition, cytotoxic DNA‐damaging agents combined with glycolysis inhibition by glucose analogue 2‐DG or HIF‐1α inhibitor showed significant antitumour effects through inducing immunogenic cell death of tumour cells [14, 15, 16]. Therefore, tumour glycolysis inhibition is expected to be effective in cancer therapy.

Signal transducer and activator of transcription 3 (STAT3) plays a carcinogenic role in a variety of malignant tumours and is positively correlated with poor prognosis [17]. Targeting STAT3 is considered a suitable intervention for treatment of tumours. Our previous studies have confirmed that blocking STAT3 signalling in HCC with STAT3 decoy‐oligodeoxynucleotide (‐ODN) could effectively inhibit the proliferation of HCC cells *in vitro* and *in vivo* [18]. Significantly, blocking STAT3 improved the antitumour immune responses against HCC and the tumour immune microenvironment, and induced antitumour immune memory [19]. Some studies showed that STAT3 inhibition could enhance the ICD of cancer cells induced by oncolytic Newcastle disease virus (NDV) and chemotherapy, in which STAT3 inhibition was used as an adjuvant therapy. NDV can induce CRT membrane translocation, the release of HMGB1, HSP70/90, and ATP in melanoma and prostate cancer cells, which could be enhanced by the STAT3 inhibitor or shRNA‐mediated depletion of STAT3 [20, 21]. The STAT3 inhibitor Stattic can increase ICD markers including CRT expression, HMGB1 and HSP70 secretion in the B16F10, and CT26 cells treated by doxorubicin [22]. In addition, STAT3 inhibition in cancer cells may stimulate the type 1 interferon response elicited by anthracyclines, resulting in an enhanced chemotherapy‐associated anticancer immune response [23, 24]. However, whether STAT3 inhibition could directly induce immunogenic death of cancer cells and the underlying molecular mechanisms have not been clarified.

In this study we tried to investigate the influences of targeting STAT3 on ICD of HCC cells *in vitro* and *in vivo*, and clarify how STAT3 inhibition improves the tumour immune microenvironment by directly disturbing the glycolysis pathway and balance between “eat me” and “don't eat me” molecules in HCC, thus providing a solid experimental basis for treatment strategies and clinical trials of HCC.

## 2 Methods

### 2.1 Cell lines and culture

The Huh7 cell line (human hepatoma cell line, purchased from the Cell Bank of Type Culture Collection of the Chinese Academy of Sciences), HepG2.2.15 cell line (an HBV genomic DNA transfected cell line, kindly supplied by Dr. Chunhong Ma, School of Basic Medical Sciences, Shandong University) and Hepa 1–6 cell line (C57BL/6‐derived hepatoma, purchased from Cell Bank of Type Culture Collection of the Chinese Academy of Sciences) were cultured in DMEM or RPMI‐1640 media (Gibco, Thermo Fisher Scientific, Waltham, MA, USA) supplemented with 10% FBS (Biological Industries, CT, USA) and 1% penicillin and streptomycin (Solarbio, Beijing, China), and were incubated at 37 °C in a 5% CO_2_ incubator.

### 2.2 Vector, lentiviruses, and transduction

The sequences of human STAT3 (GenBank accession no. NM_003150) siRNAs correspond to the coding region 1663–1681 (5′‐TGCTGACCAACAATCCCAA‐3′) were cloned into pLKO.1 plasmid and packaged in 293T cells by cotransfection with plasmid psPAX2 and pMD2G using calcium phosphate. After 48 h, the supernatant containing viral particles was collected, filtered (0.45 μm membrane), and used to infect human HCC cells in the presence of 8 μg·mL^–1^ of Polybrene (Sigma‐Aldrich, St. Louis, MS, USA).

Human STAT3 cDNA was inserted into the pCDH vector. The STAT3 mutation Y705F was constructed by a three‐step polymerase chain reaction (PCR) procedure, changing the TAC codon for tyrosine to the TTC codon for phenylalanine at the 705 site. To obtain infectious retrovirus, each construct was transfected into 293T cells with plasmid pCMV‐VSVG, pRSV‐Rev, and pMDLg/pRRE using calcium phosphate. After 48 h, the supernatants were collected and used to infect human HCC cells.

Plasmid pGFP‐V‐RS encoding mouse STAT3 shRNAs were purchased from Origen (Locus ID 20848, Origene, Rockville, MD, USA). Hepa1‐6 cells were transfected with 1 μg plasmids using Lipofectamine 2000 (Invitrogen, Carlsbad, CA, USA) in 12‐well plates and used for further experiments after 48 h.

### 2.3 Reagents

Napabucasin, STF‐31, and 2‐DG were purchased from Selleck (Selleckchem, Houston, TX, USA). Napabucasin and STF‐31 were dissolved in DMSO at 50 mm as a working stock solution, and 2‐DG was dissolved in saline. IL‐6 was purchased from PeproTech (Cranbury, NJ, USA) and stored at –80 °C. The functional grade CD47 monoclonal antibody (B6H12) was purchased from eBioscience (San Diego, CA, USA) and stored at 4 °C.

### 2.4 Annexin V staining

Apoptosis was assessed by flow cytometry using Annexin V‐FITC and PI (Sungene Biotech, Tianjin, China) following the manufacturer's instructions.

### 2.5 CCK‐8 assay

CCK‐8 (Beyotime Biotechnology, Shanghai, China) was added to each well and incubated for 1 h at 37 °C. Absorbance was measured at 450 nm using a Synergy 2 Multi‐Mode microplate spectrophotometer (BioTek).

### 2.6 ATP assay

The levels of ATP in the cultural supernatant of HCC cells were assayed by the Enhanced ATP Detection Kit (Beyotime Biotechnology).

### 2.7 Quantitative reverse transcription‐PCR (qRT‐PCR)

Total RNA from HCC cells was extracted with TRIzol reagent (Invitrogen) and synthesised to cDNAs with the SuperScript IV First‐Strand Synthesis System (Invitrogen). The LightCycler 480 SYBR Green I Master (Roche, Basel, Switzerland) was used for qRT‐PCR on a LightCycler 480 System according to the manufacturer's instruction. The relative mRNA levels were calculated using the $2^{–\Delta \Delta C_{t}}$ method. Primer sequences used are shown in Table S1.

### 2.8 Isolation of murine bone marrow‐derived DCs

Mouse bone marrow cells were isolated from 8‐week‐old C57BL/6J male mice (Beijing HFK Bioscience, Beijing, China) and cultured for 7 days in RPMI‐1640 medium (Gibco) containing 10% FBS (Biological Industries), supplemented with 20 ng·mL^–1^ murine granulocyte macrophage‐colony stimulating factor (mGM‐CSF; PeproTech), and 20 ng·mL^–1^ mIL‐4 (PeproTech) at 37 °C in a 5% CO_2_ incubator. The generated DCs were immature and their purity was determined by flow cytometry analysis of CD11c^+^ cells. Animal experiments were approved by the Animal Ethical and Welfare Committee of Shandong University (AEWC number: 18021) and were compliant with the Guide for the Care and Use of Laboratory Animals. Mice were housed in a rectangular mouse cage (area: 635 cm^2^, height: 18 cm) and were kept in a specific pathogen‐free environment under standard experimental conditions (light–dark cycle: 12 h, temperature: 20–22 °C, humidity: 50–70%) with *ad libitum* access to food and water. Five mice were housed in one cage and were cared for every day.

### 2.9 Isolation of human monocyte‐derived DCs from PBMCs

Peripheral blood mononuclear cells (PBMCs) from healthy donors were isolated by Ficoll (Solarbio) gradient centrifugation. Monocytes of the interface were collected using human CD14 MicroBeads (Miltenyi Biotec, Bergisch Gladbach, Germany), and cultured in RPMI‐1640 supplemented with 10% FBS, 100 ng·mL^–1^ hGM‐CSF (PeproTech) and 100 ng·mL^–1^ hIL‐4 (PeproTech) in a humidified atmosphere with 5% CO_2_ at 37 °C. The immature DCs were labelled with antibodies for the CD11c^+^ on the 5th day for purity analysis by flow cytometry. The collection of peripheral blood samples from healthy donors was in accordance with the Ethics Committee of Shandong University. This study was conducted under Helsinki Declaration guidelines and International Conference on Harmonisation‐Good Clinical Practices (ICH‐GCP). Written informed consent was obtained from all volunteers participating in this study.

### 2.10 Phagocytosis

Hepatocellular carcinoma cells were labelled with CFSE (Beyotime Biotechnology) and added to THP‐1‐derived macrophages labelled with CM‐Dil (Invitrogen) at a ratio of 1:1. The cells were placed in an incubator for 2 h at 37 °C and 5% CO_2_, and subsequently resuspended in phosphate‐buffered saline (PBS) and analysed by flow cytometry. The phagocytosis index was calculated as the percent of the double CFSE and CM‐Dil‐labelled cells in the THP‐1 macrophage population.

### 2.11 Western blotting

Cellular protein extracts prepared in RIPA buffer (Beyotime Biotechnology) were separated by 8% polyacrylamide gel electrophoresis and transferred to a Polyvinylidene‐Fluoride (PVDF) membrane (Millipore, Burlington, MA, USA), which was blocked with 5% fat‐free milk in PBS and then incubated with primary antibodies overnight at 4 °C. Then the PVDF membrane was incubated with HRP‐coupled goat antirabbit or mouse IgG secondary antibody (Beyotime Biotechnology). The signals were visualised with enhanced chemiluminescence (Millipore) and quantified by image‐lab software (v. 3.0; Bio‐Rad, Hercules, CA, USA). Antibodies used are shown in Table S2.

### 2.12 Coimmunoprecipitation

Cell lysates prepared in RIPA buffer were incubated with Protein A/G Magnetic Beads (Millipore) and anti‐STAT3 antibody or IgG at 4 °C overnight with rotation. After washing with PBS, proteins were collected and analysed by western blotting with anti‐STAT3 and anti‐PKR antibodies.

### 2.13 GST pull‐down

A total of 20 μg GST‐STAT3 fusion protein or GST (SignalChem, Richmond, BC, Canada) were incubated with Glutathione Magnetic Beads (PureCube, Cube Biotech, Monheim, Germany) in 500 μL PBS for 2 h at 4 °C with gentle rotation. Then 20 μg of His‐tagged PKR (Cusabio, Wuhan, China) was added to the immobilised GST‐STAT3 and GST after three washes with PBS. After being incubated overnight at 4 °C under gentle rotation, complexes were washed with PBS and collected, and analysed by western blotting with anti‐PKR and Coomassie stain.

### 2.14 Chromatin immunoprecipitation

The chromatin immunoprecipitation (ChIP) assay was performed using a kit (Millipore) according to the manufacturer's instructions. Cross‐linked immune complexes were sonicated and precipitated with IgG or anti‐p‐STAT3 antibody in the presence of Protein A/G Magnetic Beads at 4 °C overnight. DNA was extracted from the collected samples and analysed by PCR with the primers listed in Table S3.

### 2.15 Seahorse analysis of glycolysis

The XF Glycolysis Stress Test Kit (Seahorse Bioscience, North Billerica, MA, USA) was employed to evaluate glycolytic function using a Seahorse XF24 Extracellular Flux Analyser (Seahorse Bioscience) according to the manufacturer's instructions. During the experiment, the glycolysis, glycolytic capacity, and extracellular acidification rate (ECAR) were measured as 10 mm glucose, 1 μm oligomycin, and 50 mm 2‐DG were continuously loaded into the ports of the XFe‐24 sensor cartridge.

### 2.16 Orthotopic mouse model of HCC

Animal experiments were approved by the Animal Ethical and Welfare Committee of Shandong University (AEWC number: 18021) and were compliant with the Guide for the Care and Use of Laboratory Animals. Eight‐week‐old C57BL/6J male mice (Beijing HFK Bioscience) were anaesthetised by intraperitoneal injection of 10% chloral hydrate (200 mg·kg^–1^; Sinopharm Chemical Reagent) and placed on an experimental bed in a supine position. Then, a 5‐mm transverse incision was made below the xiphoid, and 2 × 10^6^ Hepa1‐6 cells suspended in 40 μL 20% Matrigel (Matrigel:PBS = 1:4) (Matrigel; Corning Incorporated, Corning, NY, USA) were injected into the left lobes of the liver. The peritoneum and skin were closed with 6‐0 sutures immediately. The living conditions of the mice were continuously monitored. After 2 weeks, the mice received 20 mg·kg^–1^ of napabucasin or vehicle by intraperitoneal injection every 2 days, with a ratio of napabucasin:Poly (ethylene glycol)‐300:Tween 80:Saline of 1:8:1:10. After a total of eight injections, the livers were harvested for further analysis. Animal experiments were approved by the Animal Ethical and Welfare Committee of Shandong University (AEWC number: 18021) and were compliant with the Guide for the Care and Use of Laboratory Animals. Mice were housed in a rectangular mouse cage (area: 635 cm^2^, height: 18 cm) and were kept in a specific pathogen‐free environment under standard experimental conditions (light–dark cycle: 12 h, temperature: 20–22 °C, humidity: 50–70%) with *ad libitum* access to food and water. Five mice were housed in one cage and were cared for every day.

### 2.17 Subcutaneous mouse model of HCC and tumour rechallenge

Animal experiments were approved by the Animal Ethical and Welfare Committee of Shandong University (AEWC number: 18021) and were compliant with the Guide for the Care and Use of Laboratory Animals. Six‐week‐old C57BL/6J male mice (Beijing HFK Bioscience) were subcutaneously injected with Hepa1‐6 cells (5 × 10^6^) in the left axilla and were monitored for visible tumour growth every 2 days. Starting on the 5th day, these mice were intraperitoneally injected with napabucasin 20 mg·kg^–1^ every 2 days. After a total of eight injections, the tumours were surgically excised for further analysis. Seven days later, the same dose of Hepa1‐6 cells (5 × 10^6^) was injected subcutaneously in the right axilla as a secondary tumour rechallenge. Tumour recurrence and growth were evaluated. Animal experiments were approved by the Animal Ethical and Welfare Committee of Shandong University (AEWC number: 18021) and were compliant with the Guide for the Care and Use of Laboratory Animals. Mice were housed in a rectangular mouse cage (area: 635 cm^2^, height: 18 cm) and were kept in a specific pathogen‐free environment under standard experimental conditions (light–dark cycle: 12 h, temperature: 20–22 °C, humidity: 50–70%) with *ad libitum* access to food and water. Five mice were housed in one cage and were cared for every day.

### 2.18 Immunofluorescence

Tumour homografts were excised after treatment, fixed with 4% paraformaldehyde, embedded in paraffin, and cut into 4‐μm‐thick sections. For immunofluorescence staining, the tissue slices were deparaffinised with xylene, successively rehydrated in 100%, 90%, 80%, and 70% ethanol, and incubated in sodium citrate buffer solution for heat‐induced epitope retrieval. After washing with PBS, the slices were blocked with goat serum and incubated with primary antibodies at 4 °C overnight. Subsequently, the slices were incubated with fluorescent conjugated antibodies for 1 h at room temperature, and observed under a fluorescence microscope (Olympus Corporation, Tokyo, Japan). Antibodies used are shown in Table S4.

### 2.19 Isolation of mononuclear cells

Hepatocellular carcinoma mouse liver and tumour tissues were harvested and disaggregated by mechanic mincing and enzymatic digestion with 0.1 mg·mL^–1^ DNAse (Roche), 1 mg·mL^–1^ type IV collagenase (Invitrogen), and 0.5% hyaluronic acid (Solarbio) for 1 h at 37 °C, then squeezed through a 200‐mesh stainless steel strainer, and the cell suspension was collected. After a brief centrifugation at 100 ***g*** for 1 min, the liver or tumour cells were at the bottom of tube, and mononuclear cells were isolated from the upper suspensions using Percoll gradient centrifugation as described before [25]. After washing with PBS, all types of cells were used for flow cytometry analysis.

### 2.20 Flow cytometric analysis

To evaluate the ICD‐related molecules, HCC cells were collected and washed with PBS, then stained with primary antibodies respectively at 4 °C for 1 h, followed by the secondary antibody Alexa Fluor 647 goat antirabbit for 40 min at 4 °C. For the analysis of immune cells, cells were harvested and washed with PBS, then stained with fluorescence‐conjugated antibodies for 40 min at 4 °C. All stained cells were acquired using a FACSCelesta or FACSCalibur flow cytometer (BD Biosciences, Franklin Lakes, NJ, USA) and analysed by flowjo software (Tree Star, San Carlos, CA, USA). Antibodies used are shown in Table S5.

### 2.21 Statistical analysis

Data analysis was performed using pasw statistics 18.0.0 (IBM‐SPSS Inc., La Jolla, CA, USA). The statistical significance between two groups was determined with unpaired Student's *t*‐test, whereas the comparison of multiple groups was carried out by one‐way ANOVA. CCK‐8 was analysed with two‐way repeated‐measures ANOVA. Tumour‐free analysis was performed using the Chi‐squared test. Survival analysis was performed using Kaplan–Meier analysis. Correlations were determined by Pearson analysis. All data were analysed for normal distribution before any statistical analyses. Data are expressed as mean ± standard deviation (SD) of three independent experiments, and considered statistically significant at *P < 0.05, **P < 0.01, and ***P < 0.001.

## 3 Results

### 3.1 Knockdown of STAT3 induces membrane translocation of ICD‐related molecules in HCC cells and promotes DC activation

**Fig. 1:**
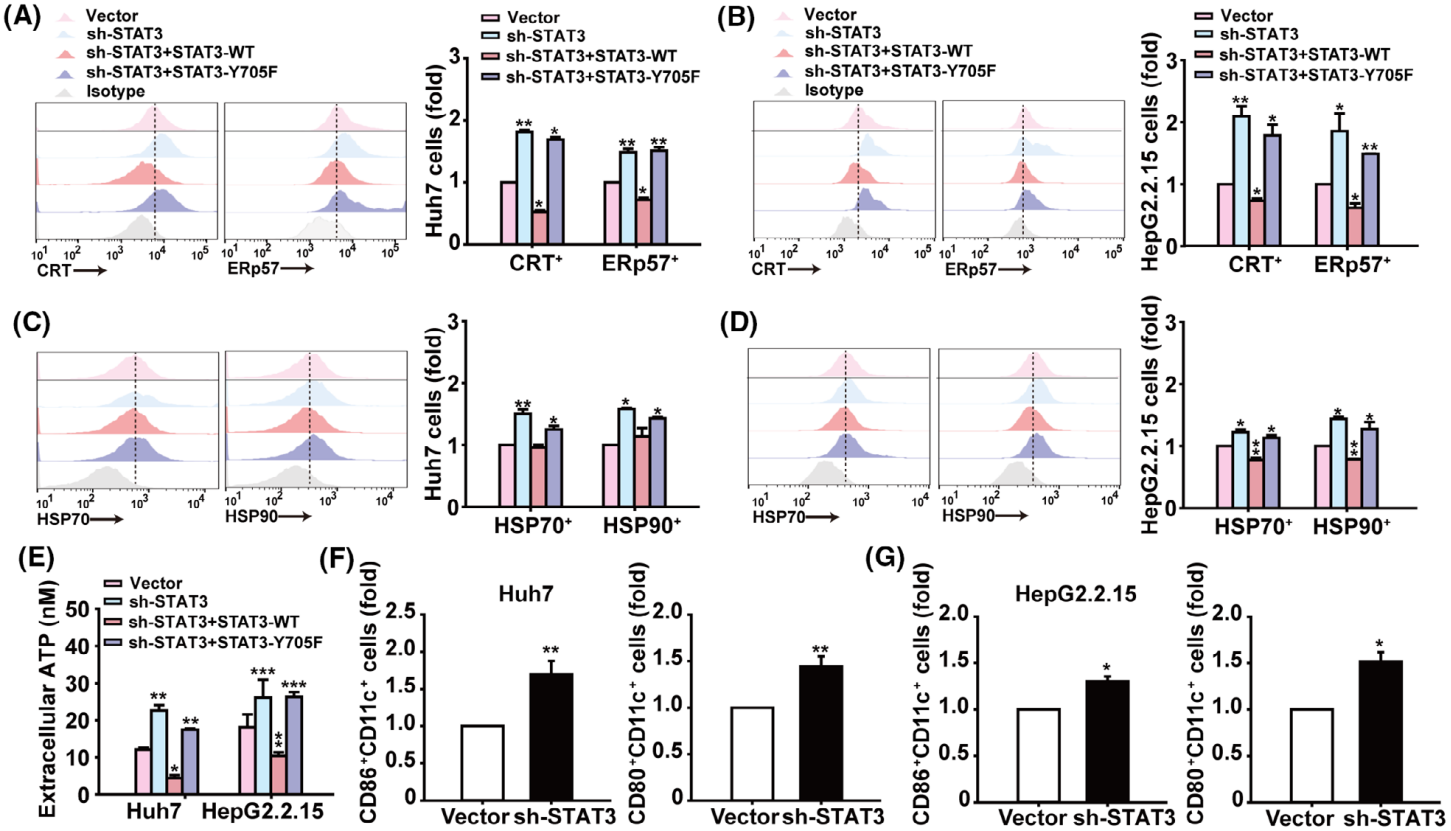
Knockdown of STAT3 expression induces membrane translocation of ICD‐related molecules in HCC cells and promotes DC activation. (A,B) Analysis of calreticulin^+^ and ERp57^+^ cells in Huh7 and HepG2.2.15 cells infected with indicated lentiviral vectors by flow cytometry. (C,D) Flow cytometry analysis of HSP70^+^ and HSP90^+^ cells in Huh7 and HepG2.2.15 cells. (E) Assessment of ATP released into culture supernatants of Huh7 and HepG2.2.15 cells. (F,G) Flow cytometry analysis of CD80^+^ and CD86^+^ cells in hDCs after coculture with Huh7 or HepG2.2.15 cells for 48 h. Data are shown as mean ± SD from three independent experiments and were analysed with unpaired Student's *t*‐test or one‐way ANOVA (*P < 0.05, **P < 0.01, and ***P < 0.001).

To understand whether STAT3 is involved in ICD of HCC cells, lentivirus carrying STAT3‐shRNA was used to interfere with the expression of STAT3, which resulted in the inhibition of the proliferation and the increase of apoptosis of HCC cells (Fig. S1A–C). In addition, we confirmed STAT3 mutation vector (STAT3‐Y705F) could not rescue the phosphorylation of STAT3 compared with wildtype STAT3 (STAT3‐WT) in Huh7 and HepG2.2.15 cells (Fig. S1D). With the increase in ATP release, we observed the expression of calreticulin, ERp57, HSP70, and HSP90 on the surface of Huh7 and HepG2.2.15 cells was significantly increased by STAT3‐knockdown, while STAT3‐Y705F rescue could not reverse this phenomenon (Fig. 1A–E). Furthermore, immature DCs derived from mononuclear cells isolated from PBMCs of healthy volunteers (Fig. S2) were coincubated with Huh7 and HepG2.2.15 cells treated with or without STAT3‐shRNA. After 48 h, compared to DCs cocultured with control HCC cells, the expression of CD80 and CD86 expression was higher on DCs coincubated with STAT3‐knockdown Huh7 or HepG2.2.15 cells (Fig. 1F,G). These data suggest that STAT3 knockdown could induce ICD of HCC cells and promote the activation of DCs.

### 3.2 Targeting STAT3 induces antitumour immune memory for HCC *in vivo*

**Fig. 2:**
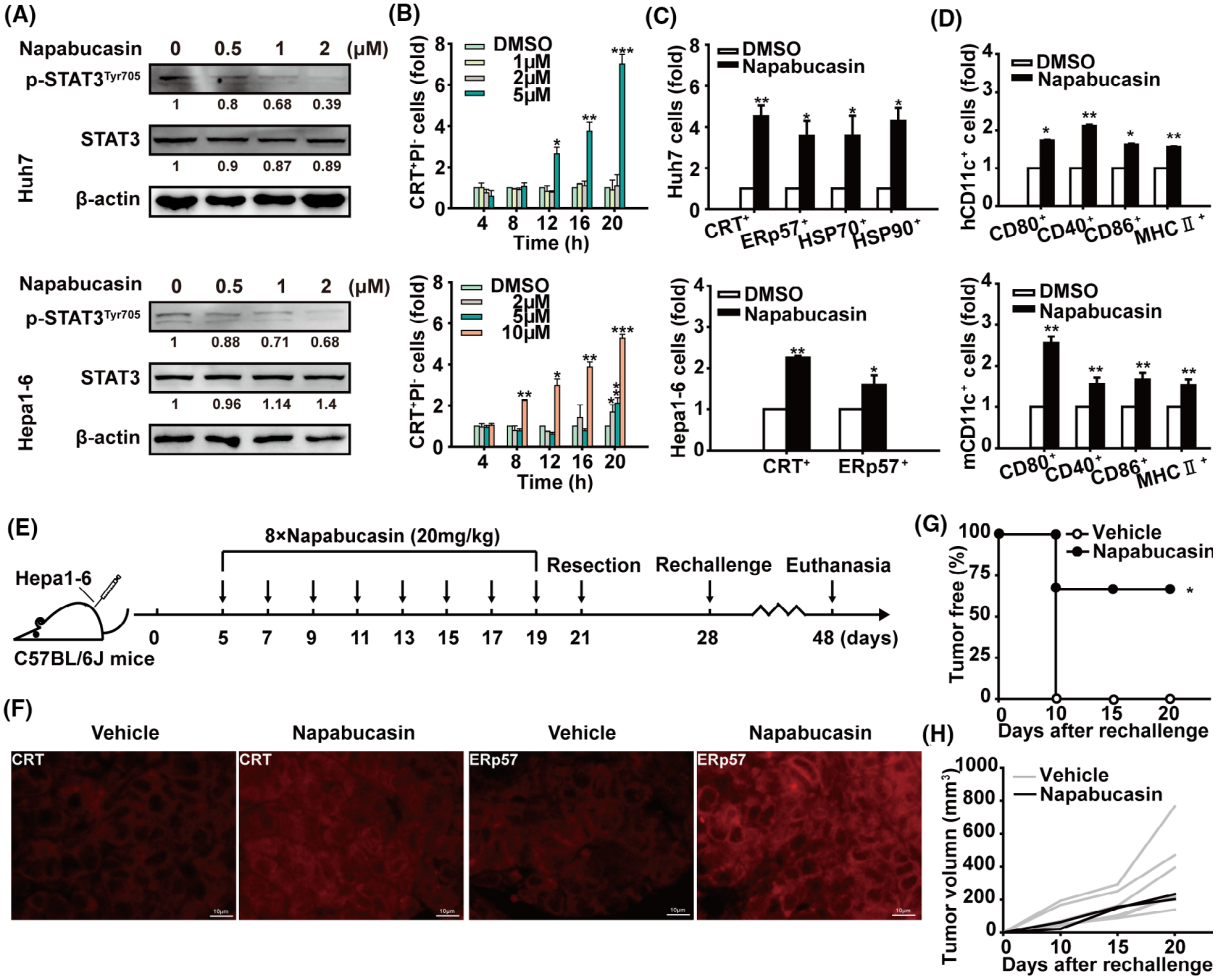
Targeting STAT3 induces immunogenic cell death of HCC *in vivo*. (A) Immunoblot showing STAT3 and p‐STAT3^Tyr705^ levels in Huh7 and Hepa1‐6 cells treated with different concentrations of napabucasin for 12 h (*n* = 3). Shown is one representative of at least three independent experiments. (B) After treatment with napabucasin or DMSO at the indicated concentrations and time, Huh7 and Hepa1‐6 cells were labelled with anti‐calreticulin antibody and PI and analysed by flow cytometry. (C) Flow cytometry analysis of calreticulin^+^, ERp57^+^, HSP70^+^, and HSP90^+^ cells in Huh7 and Hepa1‐6 cells treated with napabucasin or DMSO for 12 h. (D) Following coculture with Huh7 or Hepa1‐6 cells treated with napabucasin or DMSO for 48 h, CD80^+^, CD40^+^, CD86^+^, and MHC II^+^ cells in hDCs or mDCs were analysed by flow cytometry. Data are shown as mean ± SD from three independent experiments and were analysed with unpaired Student's *t*‐test or one‐way ANOVA (*P < 0.05, **P < 0.01, and ***P < 0.001). (E) The therapeutic schedule. C57BL/6J mice were inoculated with 5 × 10^6^ Hepa1‐6 cells in the left axilla. On day 5 postinoculation, mice were intraperitoneally injected with napabucasin (20 mg·kg^–1^) every 2 days for a total of eight injections. On day 2 after the last injection, the tumours were resected. On day 7 posttumour resection, these mice were inoculated subcutaneously with 5 × 10^6^ Hepa1‐6 cells in the right axilla. Solvent was used as vehicle. (F) The levels of calreticulin and ERp57 in tumour tissues were analysed by immunofluorescence. Scale bar = 10 μm. The tumour‐free rate (G) and tumour growth curve (H) of tumour‐rechallenged C57BL/6J mice are shown. Data represent the mean ± SD of *n* = 6 and tumour‐free analysis were analysed with the Chi‐squared test (*P < 0.05, **P < 0.01, and ***P < 0.001).

The “gold standard” to confirm the ability of a therapeutic agent to ICD currently relies on the *in vivo* vaccination assay. Subsequently, the immunogenic potential of STAT3‐inhibition was assessed in a vaccine setting. As a selective STAT3 inhibitor, napabucasin downregulated the phosphorylation of STAT3 in Huh7 and Hepa1‐6 cells (Fig. 2A). In addition, napabucasin exhibited similar effects to STAT3‐shRNA and doxorubicin in inducing the ICD of HCC cells and DC activation *in vitro* (Fig. 2B–D and Fig. S3). Six‐week‐old C57BL/6J male mice were subcutaneously injected with Hepa1‐6 cells and intraperitoneally injected with eight times napabucasin 20 mg·kg^–1^ every 2 days, then the tumours were surgically excised for further analysis. To explore whether STAT3 inhibition could induce antitumour immune memory for HCC, immunocompetent C57BL/6J mice underwent a second Hepa1‐6 cell inoculation 7 days after tumour resection (Fig. 2E). Napabucasin heightened the levels of calreticulin and ERp57 in tumour cells (Fig. 2F). Significantly, there was a 100% tumour formation on the contralateral side in the placebo group, but only 25% in the napabucasin‐treated group, in addition to slower tumour growth (Fig. 2G,H). These data demonstrated that STAT3 inhibition in HCC can promote immunogenic cell death and antitumour immune memory to prevent tumour recurrence.

### 3.3 STAT3 regulates the phosphorylation of ICD marker eIF2α

**Fig. 3:**
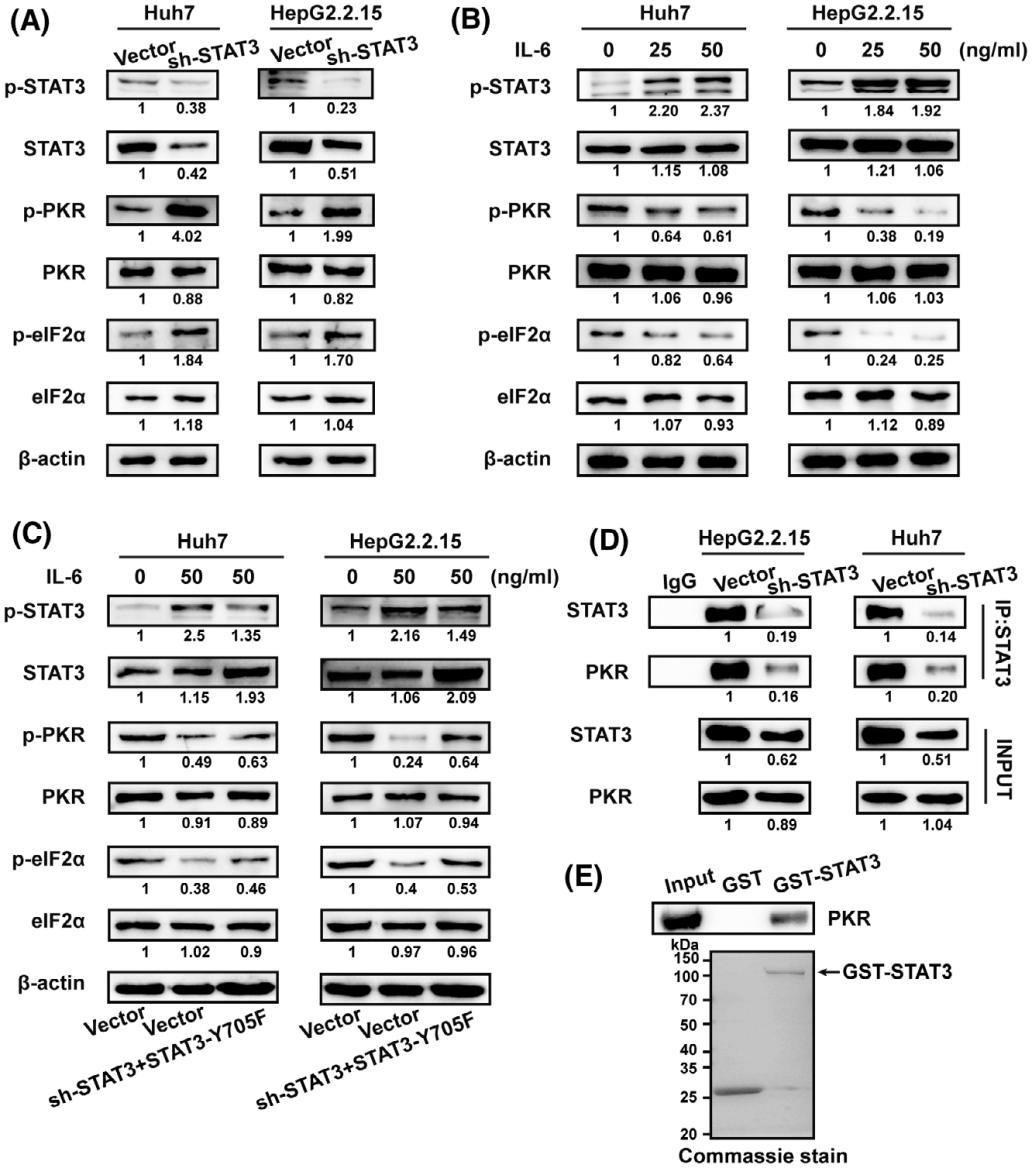
STAT3 regulates ICD marker eIF2α phosphorylation through interaction with PKR. (A) Western blot analysis of STAT3, p‐STAT3^Tyr705^, PKR, p‐PKR^Thr446^, eIF2α, and p‐eIF2α^Ser51^ expression in Huh7 and HepG2.2.15 cells infected with lentiviral STAT3‐shRNA vector or lentiviral vector. (B) Western blot analysis of STAT3, p‐STAT3^Tyr705^, PKR, p‐PKR^Thr446^, eIF2α, and p‐eIF2α^Ser51^ expression in Huh7 and HepG2.2.15 cells stimulated with IL‐6 at the indicated concentrations for 2 h. (C) Western blot analysis of STAT3, p‐STAT3^Tyr705^, PKR, p‐PKR^Thr446^, eIF2α, and p‐eIF2α^Ser51^ expression in Huh7 and HepG2.2.15 cells infected with indicated lentiviral vectors after stimulation with IL‐6. (D) Coimmunoprecipitation with STAT3 antibody. Huh7 and HepG2.2.15 cell extracts were incubated with anti‐STAT3 antibody and Protein A/G Magnetic Beads. Following incubation, PKR was detected by western blot to verify the protein binding. (E) GST pull‐down assay of the interaction between STAT3 and PKR. Western blotting analysis of PKR binding to purified GST or GST‐STAT3 fusion protein using PKR antibody (top). GST or GST‐STAT3 fusion protein was visualised by Coomassie blue staining (bottom). *N* = 3. Shown is one representative of at least three independent experiments.

Eukaryotic translation initiation factor 2α (eIF2α) kinase‐induced phosphorylation of eIF2α is necessary for calreticulin translocation [26]. Here we found that knockdown of STAT3 significantly upregulated the expression and phosphorylation of eIF2α kinase PKR in Huh7 and HepG2.2.15 cells, accompanied by increased expression and phosphorylation of eIF2α (Fig. 3A). By contrast, upon IL‐6 induced phosphorylation of STAT3, the levels of p‐PKR and its downstream molecule p‐eIF2α were significantly decreased (Fig. 3B). In addition, STAT3‐Y705F transfection could not reverse STAT3 knockdown‐mediated and the increase of p‐PKR and its downstream molecule p‐eIF2α in Huh7 and HepG2.2.15 cells under IL‐6 treatment (Fig. 3C). The SH2 domain of STAT3 interacts with the C terminal of PKR to form a complex, regulating the level of free and phosphorylated PKR, as well as the phosphorylation of eIF2α in U2OS cells [27]. IP and GST pull‐down experiments showed that STAT3‐PKR complex formation was clearly inhibited in STAT3‐silenced Huh7 and HepG2.2.15 cells (Fig. 3D,E). Therefore, STAT3 regulates eIF2α activation via the formation of STAT3‐PKR complexes in HCC cells.

### 3.4 STAT3 directly regulates “don't eat me” molecule CD47 in HCC cells

**Fig. 4:**
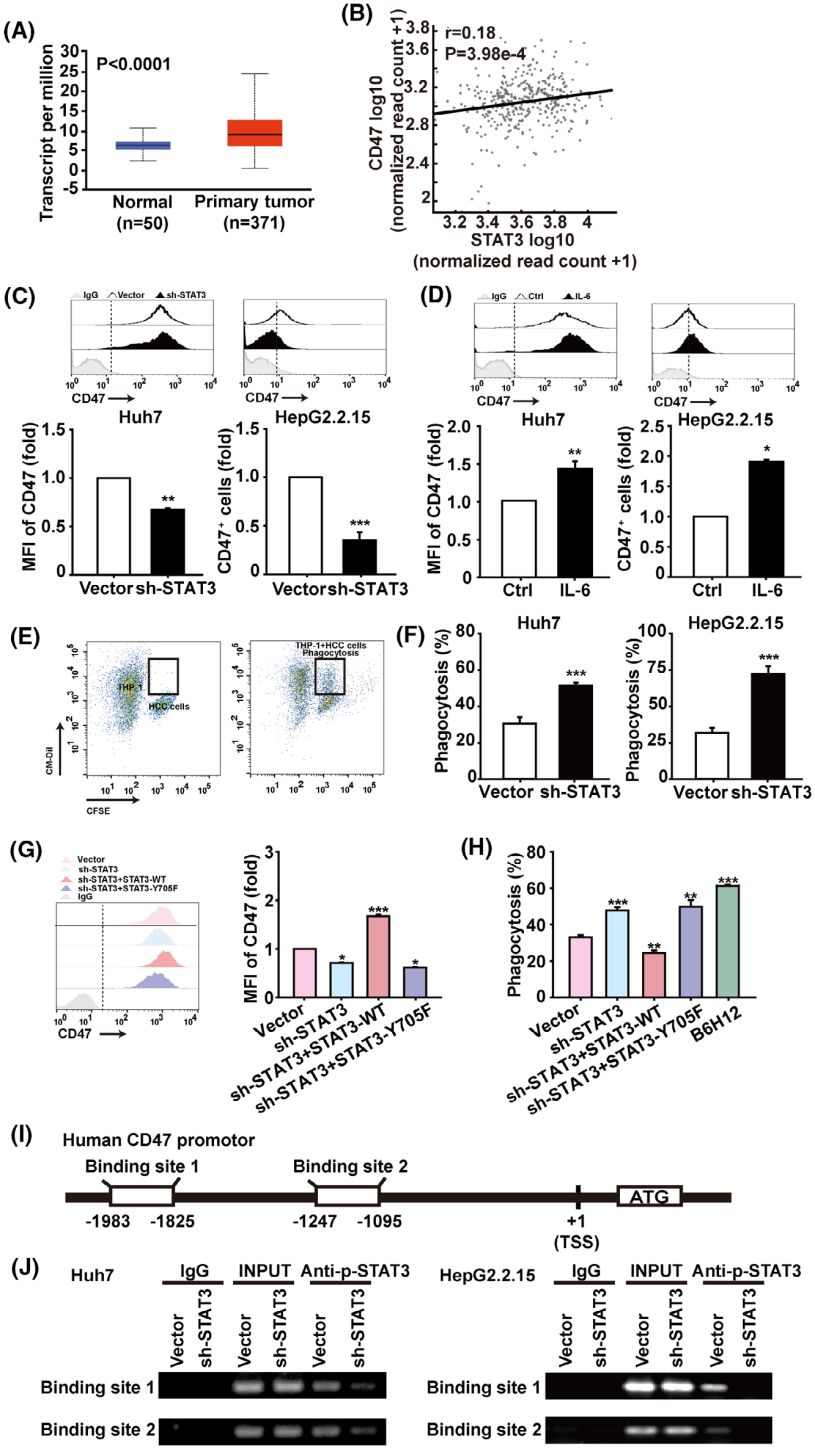
STAT3 directly regulates “don't eat me” signal CD47 in HCC cells. (A) TCGA database analysis of CD47 expression in HCC patients based on sample types through the UALCAN database. (B) Validation of gene expression correlation between STAT3 and CD47 in HCC patients through the AIPuFu database. Data from the TCGA database. Correlations were determined by Pearson analysis. (C) Huh7 and HepG2.2.15 cells were infected with lentiviral STAT3‐shRNA or control vectors, and CD47 expressions were analysed by flow cytometry. (D) Flow cytometry analysis of CD47 expression in Huh7 and HepG2.2.15 cells stimulated with 50 μm IL‐6 for 4 h. (E) CM‐Dil‐labelled macrophages derived from THP‐1 cells were cocultured with CFSE‐labelled Huh7 or HepG2.2.15 cells for 2 h, then the phagocytosis was assessed by flow cytometry. (F) The statistical results of phagocytosis. Data are shown as mean ± SD from three independent experiments and were analysed with unpaired Student's *t*‐test (*P < 0.05, **P < 0.01, and ***P < 0.001). (G) Flow cytometry analysis of CD47^+^ cells in Huh7 cells. (H) The statistical results of phagocytosis. Antihuman CD47 antibody (B6H12) was used to block CD47 on the surface of HCC cells at the 2 μg·mL^–1^ for 2 h coincubation at 37 °C. Data are shown as mean ± SD from three independent experiments and were analysed with one‐way ANOVA (*P < 0.05, **P < 0.01, and ***P < 0.001). (I) Schematic representation shows two candidate STAT3 binding sites on human CD47 gene promoter. (J) ChIP assays were performed to identify the binding of STAT3 to the CD47 promoter in HCC cells. Human anti‐p‐STAT3 antibody or control IgG were used for immunoprecipitation with DNA from Huh7 and HepG2.2.15 cells. The immunoprecipitates were then amplified by PCR using primers targeting CD47, *n* = 3. Shown is one representative of at least three independent experiments.

The balance between the “don't eat me” and “eat me” signalling influences the recognition and phagocytosis of tumour cells by DCs and macrophages, as well as the antitumour immune responses. As the “don't eat me” signalling molecule, CD47 prevents macrophage‐mediated phagocytosis of tumour cells. TCGA database analysis showed that the expression of CD47 is increased and positively correlated with STAT3 in HCC (Fig. 4A,B). We found that STAT3 knockdown markedly reduced CD47 levels on Huh7 and HepG2.2.15 cells (Fig. 4C), while treatment with the STAT3 activator IL‐6 significantly increased the cell‐surface expression of CD47 (Fig. 4D). Interestingly, after coincubation of CFSE‐labelled Huh7 and HepG2.2.15 cells with THP‐1‐induced macrophages for 2 h, flow cytometry showed that STAT3 knockdown could promote macrophage‐mediated phagocytosis of HCC cells (Fig. 4E,F). In addition, STAT3‐WT overexpression markedly increased CD47 levels on Huh7 cells, while STAT3‐Y705F overexpression could not rescue the STAT3 knockdown‐mediated CD47 decrease (Fig. 4G). The antihuman CD47 antibody B6H12 that could effectively block CD47‐epitope on the surface of HCC cells [28, 29] greatly increased macrophage‐mediated phagocytosis of Huh7 cells, which was similar to STAT3‐knockdown. However, STAT3‐Y705F overexpression could not inhibit STAT3 knockdown‐induced augmentation of macrophage‐mediated phagocytosis of HCC cells compared to wildtype STAT3 (Fig. 4H). To verify whether STAT3 could directly control CD47 expression in HCC cells, we collected the promoter sequence of CD47 from the JASPAR database, and predicted four candidate binding sites of STAT3. ChIP and sequencing analyses demonstrated that STAT3 could directly bind to the regions from –1983 to –1825 bp and from –1247 to –1095 bp in the CD47 promoter (Fig. 4I,J and Fig. S4). These results thus show that STAT3 directly controls the expression of CD47 on the surface of HCC cells, preventing macrophage–mediated phagocytosis and antitumour immunity.

### 3.5 STAT3 inhibits ICD via directly regulating glycolysis in HCC cells

**Fig. 5:**
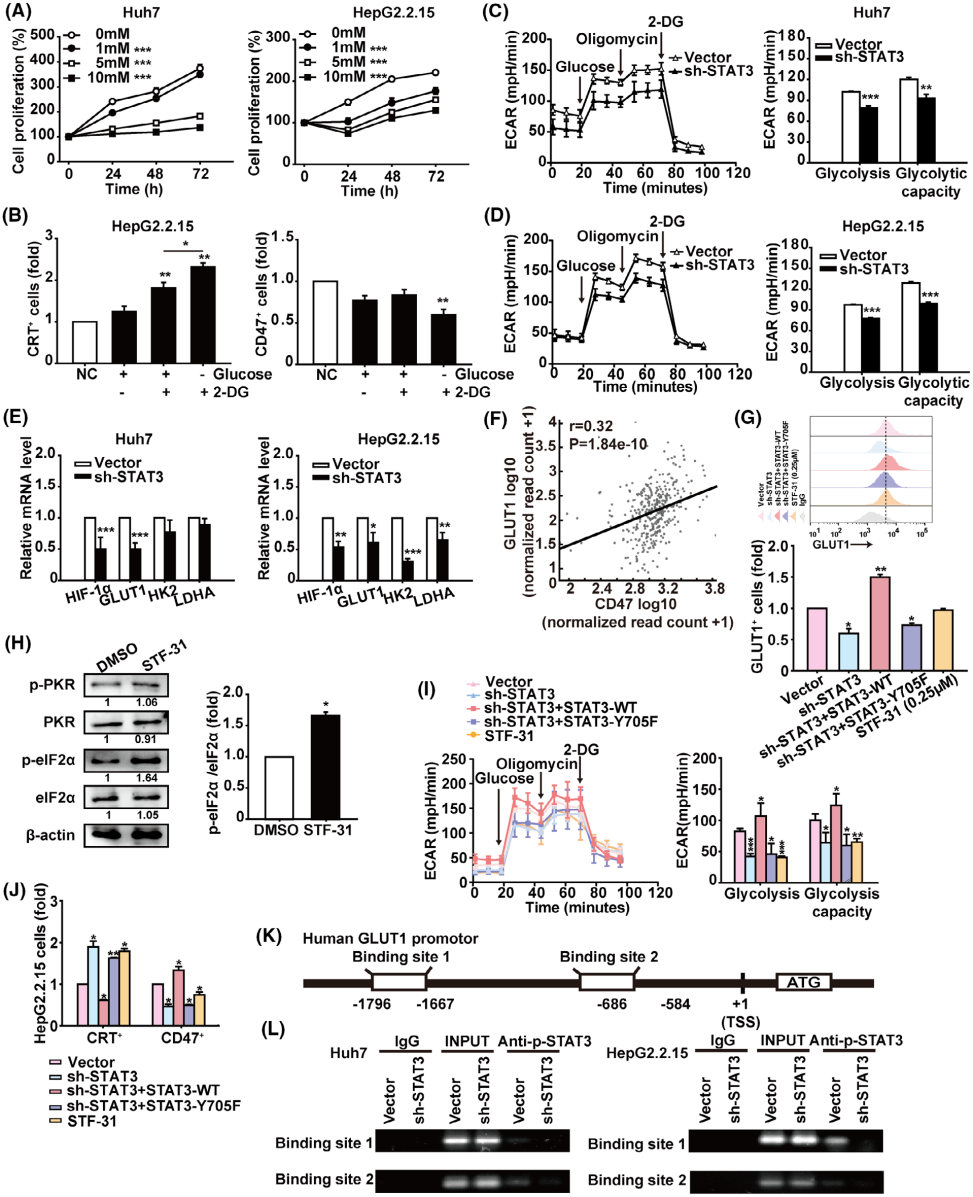
STAT3 regulates the glycolysis of HCC cells. (A) CCK‐8 assay of Huh7 and HepG2.2.15 cells treated with saline or the indicated concentrations of 2‐DG for 24, 48, and 72 h. (B) HepG2.2.15 cells were cultured in glucose‐free medium for 6 h to implement glucose starvation, then replaced with fresh medium containing glucose or/and 2‐DG for another 12 h. These cells were harvested for analysis of calreticulin and CD47 levels by flow cytometry. (C,D) Huh7 and HepG2.2.15 cells were infected with lentiviral STAT3‐shRNA vector or control vector for 48 h. The ECAR value was automatically recorded and calculated by the Seahorse XF‐24 analyser in Huh7 and HepG2.2.15 cells. (E) The mRNA levels of HIF1α, GLUT1, HK2, and LDHA in Huh7 and HepG2.2.15 cells infected with lentiviral STAT3‐shRNA vector or control vector were analysed by qRT‐PCR. Data were normalised to β‐actin and are shown as mean ± SD from three independent experiments. Data are shown as mean ± SD from three independent experiments and were analysed with unpaired Student's *t*‐test or one‐way ANOVA (*P < 0.05, **P < 0.01, and ***P < 0.001). (F) AIPuFu database analysis of the correlation between GLUT1 and CD47 in HCC from the TCGA database. Correlations were determined by Pearson analysis. (G) Flow cytometry analysis of GLUT1^+^ cells in HepG2.2.15 cells treated as indicated. (H) Western blot analysis of PKR, p‐PKR^Thr446^, eIF2α, and p‐eIF2α^Ser51^ expression in HepG2.2.15 cells treated with STF‐31 (0.25 μm) for 12 h. The grey scale of protein bands was quantified by imagej (National Institutes of Health, Bethesda, MD, USA). Data are shown as mean ± SD from three independent experiments and were analysed with unpaired Student's *t*‐test (*P < 0.05). (I) The ECAR value of HepG2.2.15 cells treated as indicated. The statistical results of glycolysis and glycolysis capacity are shown. Data are shown as mean ± SD from three independent experiments and were analysed with one‐way ANOVA (*P < 0.05, **P < 0.01, and ***P < 0.001). (J) Analysis of calreticulin^+^ and CD47^+^ cells in HepG2.2.15 cells treated as indicated by flow cytometry. Data are shown as mean ± SD from three independent experiments and were analysed with one‐way ANOVA (*P < 0.05 and **P < 0.01). (K) Schematic representation shows two candidate STAT3 binding sites on human GLUT1 gene promoter. (L) ChIP assays were performed to evaluate binding of STAT3 to the GLUT1 promoter in HCC cells. Human anti‐STAT3 antibody or control human IgG was used for immunoprecipitation with DNA from Huh7 and HepG2.2.15 cells. The immunoprecipitates were then amplified by PCR using primers targeting GLUT1. Shown is one representative of at least three independent experiments.

Rapid proliferation of tumour cells is closely related to abnormal glucose metabolism, consuming a large amount of glucose for aerobic glycolysis. Here we observed that the glucose analogue 2‐DG could inhibit the proliferation of Huh7 and HepG2.2.15 cells in a time‐ and concentration‐dependent manner (Fig. 5A). Furthermore, 2‐DG treatment resulted in a calreticulin increase but CD47 decrease on HepG2.2.15 cells glucose‐starved for 6 h and then allowed (or not) to recover (Fig. 5B). Subsequently, the TCGA database showed an elevated expression of key glycolysis‐related molecules such as HIF‐1α, GLUT1, and HK2 in HCC, associated with short survival of HCC patients. Although we found no significant difference in LDHA expression between normal and tumour tissues, HCC patients with high LDHA levels showed a decreased survival time (Fig. S5A,B). Moreover, STAT3 was positively correlated with HIF‐1α, GLUT1, HK2, and LDHA (Fig. S5C). Significantly, STAT3 knockdown inhibited glycolysis and maximum glycolysis capacity of Huh7 and HepG2.2.15 cells (Fig. 5C,D), accompanied with downregulation of HIF‐1α, GLUT1, HK2, and LDHA (Fig. 5E). Similar results were shown in Huh7 and HepG2.2.15 cells treated with the STAT3 inhibitor napabucasin (Fig. S6). These data indicate that STAT3 regulates the glycolysis that is associated with the proliferation and ICD of HCC cells.

GLUTs carry out the first critical step in the glycolysis pathway, transporting glucose into tumour cells. TCGA database analysis showed the expression of GLUT1 is increased and positively correlated with CD47 in HCC (Fig. 5F). We found STAT3 knockdown markedly reduced GLUT1 levels in HepG2.2.15 cells (Fig. 5G), and the GLUT1 selective inhibitor STF‐31 [30, 31] increased the level and activation of eIF2α (Fig. 5H). Similar to STF‐31, STAT3 knockdown greatly decreased glycolysis and maximum glycolysis capacity of HepG2.2.15 cells (Fig. 5I), and suppressed the expression of calreticulin and CD47 (Fig. 5J), which could not be rescued by STAT3‐Y705F as wildtype STAT3. To confirm whether STAT3 regulated glycolysis directly via GLUT1 in HCC cells, first we analysed the GLUT1 promoter sequence through the JASPAR database, and predicted three candidate binding sites for STAT3. Then ChIP assays proved that STAT3 could directly bind to the regions from –1796 to –1667 bp and from –686 to –584 bp in the GLUT1 promoter (Fig. 5K,L and Fig. S7). These results demonstrated that STAT3 is involved in the glycolysis of HCC cells, which dampens the induction of ICD of HCC.

### 3.6 STAT3 inhibition improves the tumour immune microenviroment in HCC mouse models

**Fig. 6:**
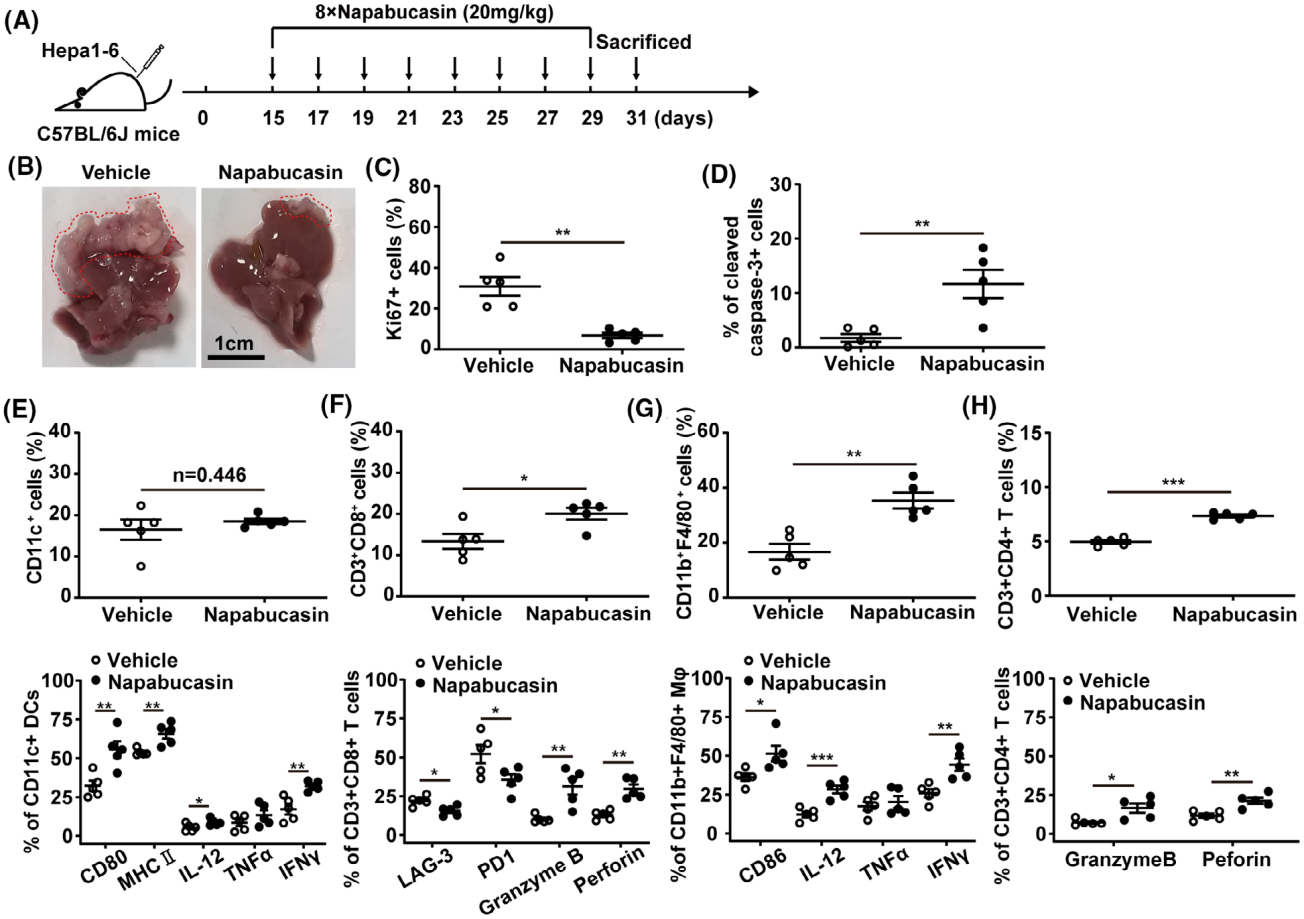
Napabucasin triggers the antitumour immune response in the orthotopic mouse model of HCC. C57BL/6J mice were inoculated with 2 × 10^6^ Hepa1‐6 cells in the left lobe of the liver. On day 15 postinoculation, mice were intraperitoneally injected with napabucasin (20 mg·kg^–1^) every 2 days for a total of eight injections. Solvent was used as vehicle. Liver cells and mononuclear cells were isolated and analysed by flow cytometry. (A) The therapeutic schedule. (B) The liver from orthotopic liver transplantation mice, and the red line shows grafts after growth. Scale bar = 1 cm. The frequency of Ki67^+^ (C) and cleaved caspase‐3^+^ cells (D) in livers was assessed by flow cytometry. (E) The proportion of CD11c^+^ DCs (up), CD80^+^, MHC II^+^, IL‐12^+^, IFNγ^+^ and TNFα^+^ DCs (bottom) DCs infiltrated in liver was analysed by flow cytometry. (F) The proportion of CD3^+^CD8^+^ T cells (up), LAG‐3^+^, PD1^+^, Granzyme B^+^ and Perforin^+^ CD8^+^ T cells (bottom) in liver was analysed by flow cytometry. (G) The frequency of CD11b^+^F4/80^+^ macrophages (up) and CD86^+^, IL‐12^+^, IFNγ^+^ and TNFα^+^ macrophages in liver was assessed by flow cytometry. (H) The proportion of CD3^+^CD4^+^ T cells (up), and Granzyme B^+^ and Perforin^+^ CD4^+^ T cells (bottom) was analysed by flow cytometry. Mφ, macrophage. Data represent the mean ± SD of *n* = 5 and were analysed with one‐way ANOVA (*P < 0.05, **P < 0.01, and ***P < 0.001).

**Fig. 7:**
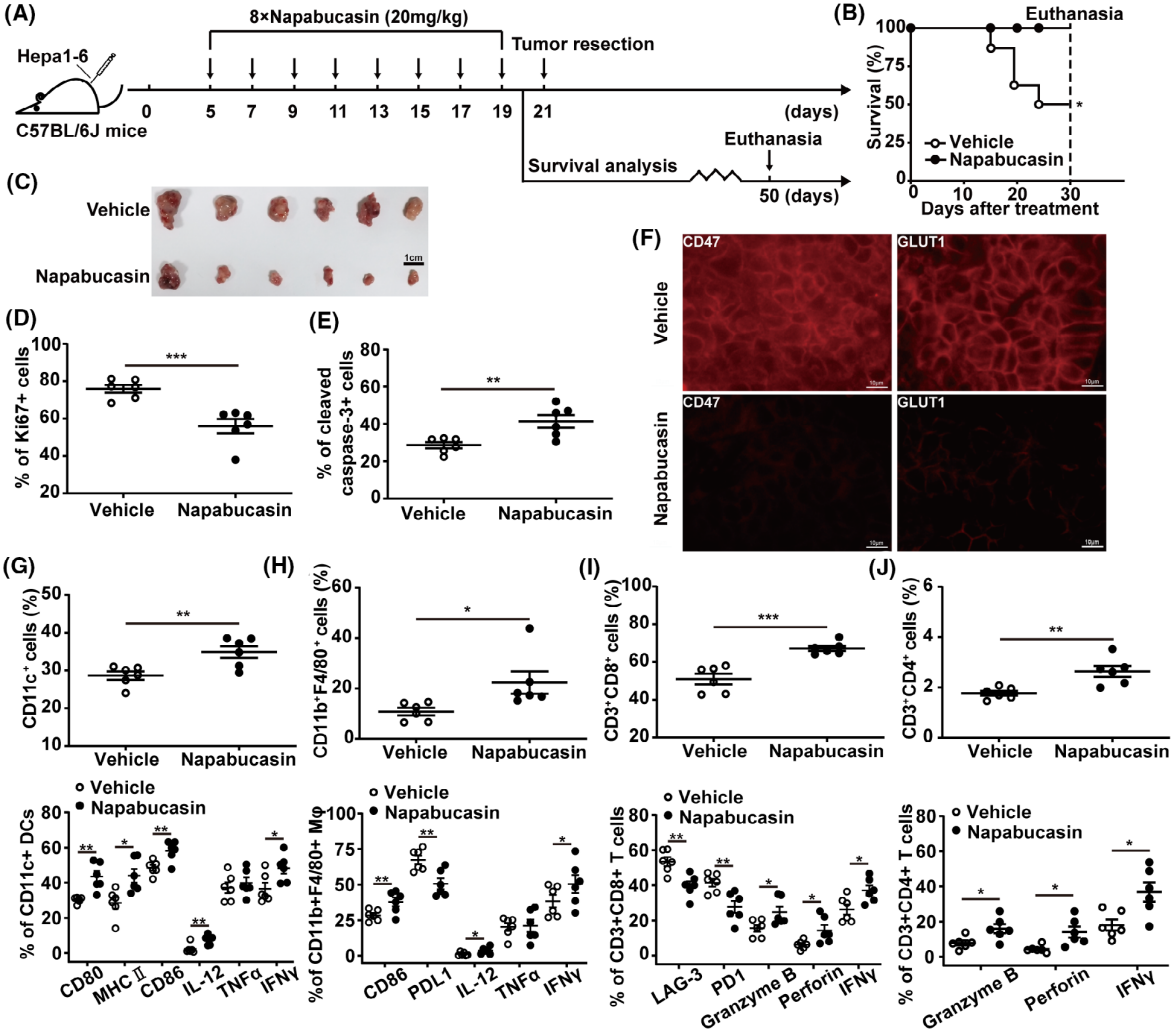
Napabucasin induces antitumour immunity of HCC in the subcutaneous mouse model. (A) The therapeutic schedule. C57BL/6J mice were inoculated with 5 × 10^6^ Hepa1‐6 cells in the left axilla. On day 5 postinoculation, mice were intraperitoneally injected with napabucasin (20 mg·kg^–1^) every 2 days for a total of eight injections. Solvent was used as vehicle. (B) The overall survival was monitored for 30 days after treatment. Survival analysis was performed using Kaplan–Meier analysis. On day 21 postinoculation, tumour tissues (scale bar = 1 cm) were collected from mice treated with napabucasin as described above (C), and the frequency of Ki67^+^ (D) and cleaved caspase‐3^+^ cells (E) in tumours was assessed by flow cytometry. (F) The levels of CD47, and GLUT1 in tumour tissues were analysed by immunofluorescence. Scale bar = 10 μm. The proportion of infiltrated CD11c^+^ DCs (up), and CD80^+^, MHC II^+^, CD86^+^, IL‐12^+^, IFNγ^+^ and TNFα^+^ DCs (bottom) (G), CD11b^+^F4/80+ macrophages (up), and CD86^+^, PDL1^+^, IL‐12^+^, IFNγ^+^ and TNFα^+^ macrophages (bottom) (H), CD3^+^CD8^+^ T cells (up), and LAG‐3^+^, PD1^+^, Granzyme B^+^, Perforin^+^ and IFNγ^+^ CD8^+^ T cells (bottom) (I), CD3^+^CD4^+^ T cells (up), and Granzyme B^+^, Perforin^+^ and IFNγ^+^ CD4^+^ T cells (bottom) (J) was analysed by flow cytometry. Data represent the mean ± SD of *n* = 6 and were analysed with one‐way ANOVA (*P < 0.05, **P < 0.01, and ***P < 0.001).

We next evaluated the antitumour effect of STAT3 inhibition in a liver orthotopic transplantation mouse model with Hepa1‐6 cells. Starting on day 15 after tumour inoculation, the mice were intraperitoneally injected with 20 mg·kg^–1^ napabucasin or placebo every 2 days for eight consecutive times (Fig. 6A). As shown in Fig. 6B, napabucasin significantly suppressed tumour growth, manifested as the reduction of tumour nodules and no significant influence on liver weight (Fig. S8). Meanwhile, napabucasin markedly reduced Ki67 and increased cleaved caspase‐3 expression in liver cells (Fig. 6C,D). Although the number of infiltrated DCs in the liver did not show a noticeable change, the expression of CD80, MHCII, IL‐12, and IFNγ in DCs was significantly increased by napabucasin treatment (Fig. 6E). In addition, napabucasin enhanced the infiltration of CD8^+^ T cells, concomitantly downregulated immunosuppressive molecules such as LAG‐3 and PD‐1, and upregulated cytotoxicity factors such as Granzyme B and Perforin (Fig. 6F). The infiltration and CD86, IL‐12, IFNγ expression of CD11b^+^F4/80^+^ macrophages were also significantly increased in the livers of napabucasin‐treated mice (Fig. 6G). Further, napabucasin treatment augmented the infiltration of CD4^+^ T cells with upregulation of Granzyme B and Perforin (Fig. 7H).

Subsequently, a subcutaneous homograft mouse model of Hepa1‐6 cells was established to further verify whether STAT3 inhibition could induce ICD of HCC *in vivo* (Fig. 7A). Remarkably, the phosphorylation of STAT3 was reduced in tumour cells from napabucasin‐treated mice compared to that in vehicle‐treated mice (Fig. S9). And napabucasin prolonged the overall survival of HCC‐bearing mice (Fig. 7B), markedly delayed the tumour growth (Fig. 7C and Fig. S10) with the reduction of Ki67, and the increase of cleaved caspase‐3 in tumour cells (Fig. 7D,E). Furthermore, napabucasin restrained the levels of CD47 and GLUT1 (Fig. 7F). Significantly, napabucasin treatment promoted the infiltration of DCs in tumour tissues, concomitantly increased the expression of antigen presentation‐associated molecules such as CD80, CD86, and MHC II, and the inflammatory cytokines IL‐12 and IFNγ (Fig. 7G), and simultaneously augmented the infiltration of macrophages with upregulation of CD86, IL‐12, IFNγ, and downregulation of PD‐L1 (Fig. 7H). Tumour infiltration of CD8^+^ T cells was also enhanced by napabucasin treatment, and exhibited lower levels of LAG‐3, PD‐1 and high levels of Granzyme B, Perforin, IFNγ in the napabucasin‐treated mice than in the control group (Fig. 7I). Moreover, napabucasin treatment augmented the infiltration of CD4^+^ T cells with upregulation of Granzyme B, Perforin, and IFNγ (Fig. 7J). Meanwhile, napabucasin treatment significantly downregulated PD‐L1 and upregulated CD95 in the tumours of HCC mouse models (Fig. S11), further enhancing the anti‐HCC efficiency.

Clinically, the noticeable symptoms of HCC are not obvious at the initial stage. Once the patients show the clinical symptoms of HCC, most of them converted to the middle or advanced stage [32, 33]. Thus, we also evaluated the anti‐HCC effect of napabucasin in mice bearing a large tumour load (Fig. S12A). Although tumour growth was not significantly inhibited by napabucasin (data not shown), LAG‐3^+^ and Tim‐3^+^ CD8^+^ T cells were significantly reduced, while the expression of IFNγ was significantly increased (Fig. S12B). Similar results were seen in infiltrated NK cells (Fig. S12C). These data demonstrated that STAT3 inhibition in HCC can improve the immune microenvironment, promoting the antitumour immune responses.

## 4 Discussion

**Fig. 8:**
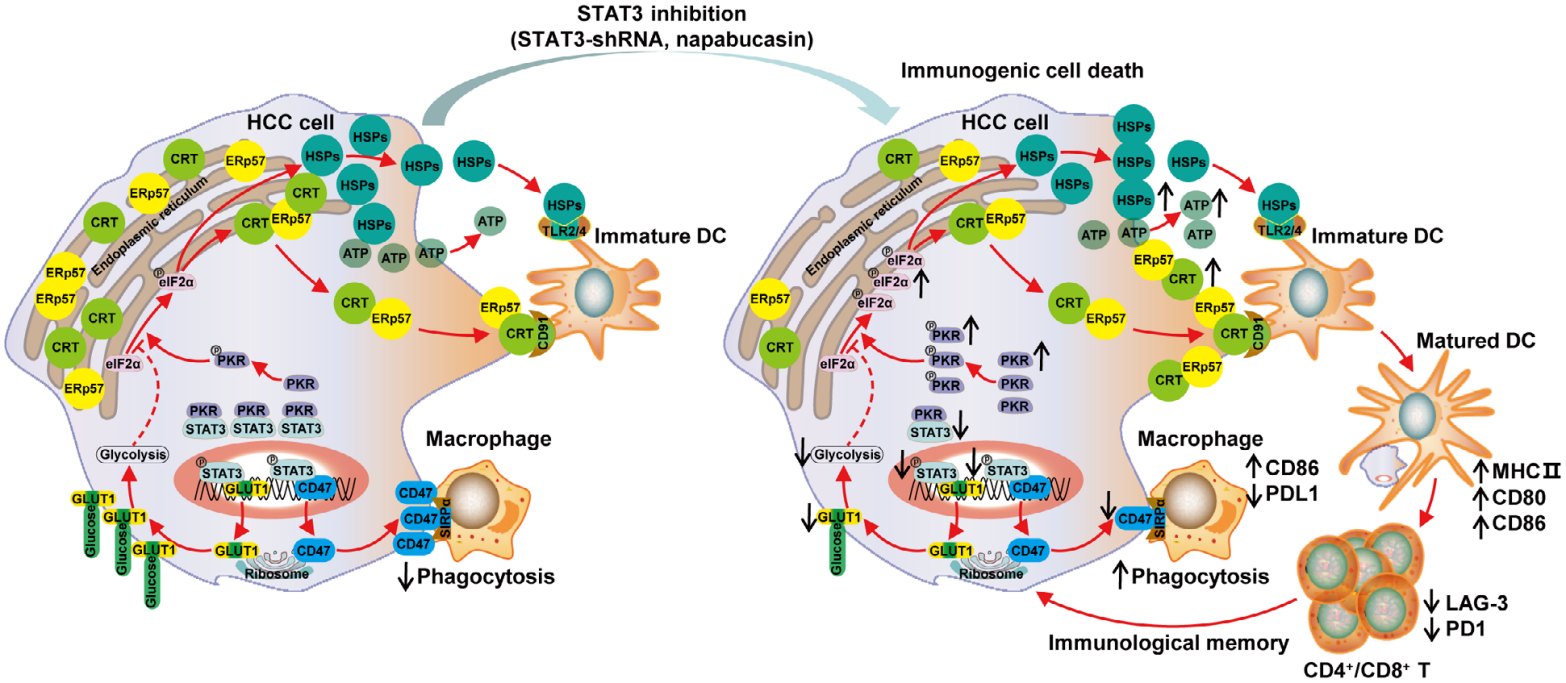
STAT3 inhibition induces ICD and improves the tumour immune‐environment in HCC. Targeting inhibition of STAT3 could induce ICD of HCC cells, which manifested as “eat me” molecules such as calreticulin, ERp57, and HSPs translocated to the HCC cell surface while the “don't eat me” molecule CD47 significantly decreased. This enhanced the recognition and phagocytosis of HCC cells by DCs and macrophages. In parallel, the important antitumour effector CD8^+^ T cells, with low expression of checkpoint molecules such as LAG‐3 and PD‐1, accumulated in tumour tissues. The underlying mechanism involves the disruption of STAT3‐mediated direct regulation of CD47 and glycolysis via GLUT1 in HCC cells.

STAT3 is constitutively activated in HCC cells and persistently promotes tumour progression through direct regulation of oncogenic gene expression. However, STAT3 also paves the way for tumour growth through immunosuppression [34]. STAT3 signalling molecules hinder the conversion of cold to hot tumours by regulating immunosuppressive molecule secretion and immunosuppressive cell functions [35]. In this study we found that targeting STAT3 could induce ICD of HCC cells, which manifested as translocation of “eat me” molecules such as calreticulin to the cell surface, while exposure of the “don't eat me” molecule CD47 significantly decreased. STAT3 inhibition enhanced the recognition and phagocytosis of HCC cells by macrophages, and diminished HCC‐induced disturbance of DC maturation and activation. Significantly, targeting STAT3 induced anti‐HCC immune memory and accumulation of the important antitumour effector CD8^+^ T cells in tumour tissues, which expressed low levels of checkpoint molecules such as LAG‐3 and PD‐1. The underlying mechanism involves the disruption of STAT3‐mediated direct regulation of CD47 and glycolysis via GLUT1 in HCC cells (Fig. 8).

In the early stages of ICD, the “don't eat me” and “eat me” signals are in a trade‐off state, and tumour cells can thus be effectively recognised and phagocytosed by DCs and macrophages, triggering the antitumour immune responses [36]. ICD therefore constitutes an effective pathway to activate the immune system against cancer, which in turn determines the long‐term success of anticancer therapies. Here, knockdown of STAT3 altered calreticulin, ERp57, HSPs, and CD47 levels on the cell surface as well as the release of soluble mediators such as ATP, which stimulate the tumour antigen‐presentation ability of DCs and macrophages. And extracellular ATP also as a “find me” signal acts on purinergic receptors of metabotropic P2Y2, which could recruit the ATP‐mediated chemotaxis of DCs into the tumour site [37]. Interestingly, STAT3 directly bound to the promoter of CD47 and regulated its gene transcription. Therefore, STAT3 modulates ICD of HCC by regulating the membrane translocation of the ICD‐related molecules calreticulin and ERp57, the release of ATP to extracellular and through direct control of CD47 expression.

In response to the ICD inducers anthracyclines and oxaliplatin, patients with breast and colorectal cancer acquire a favourable therapeutic response manifesting increased numbers of cytotoxic CD8^+^ T cells within the tumour [38, 39]. Chemotherapy‐driven ICD relies on the induction of endoplasmic reticulum (ER) stress or alteration in ER homeostasis to promote the eIF2α phosphorylation‐dependent membrane translocation of ER chaperones calreticulin and ERp57 [40]. During ICD, eIF2α phosphorylation induced by eIF2α kinases is necessary for calreticulin translocation [41]. The eIF2α kinases are highly conserved serine/threonine protein kinases, which are widely expressed in mammalian tissues and participate in various stress responses by regulating the activity of eIF2α [42]. In mammalian cells, distinct stress conditions activate different kinases (PERK, PKR, GCN2, and HRI) that converge on phosphorylating a unique serine in eIF2α [43]. PKR can be activated by ICD inducers, including anthracyclines, oxaliplatin, and radioactive irradiation, and thereby promotes the ICD of tumour cells [44]. The SH2 domain of STAT3 could interact with the C‐terminal domain of PKR to regulate the levels of phosphorylated and unphosphorylated kinase, thus influencing the phosphorylation of eIF2α and autophagy in U2OS cells [27]. We found STAT3 knockdown significantly upregulated the expression and phosphorylation of eIF2α as well as PKR in HCC cells. In addition, we observed that STAT3 controlled the levels and phosphorylation of PKR by forming a reversible complex with it, thereby regulating the phosphorylation of eIF2α.

Aerobic glycolysis is the main metabolic pathway of tumour cells, which enhances their proliferation capacity and adaptability to the microenvironment [45, 46]. HIF‐1α, a key regulator of the Warburg effect, modulates the transcription of hypoxic reactivity genes and participates in the signal transduction process during hypoxia, in order to adapt to the hypoxic conditions and maintain homeostasis of the internal environment [47]. STAT3 directly regulates HIF‐1α by binding to its promoter, which affects the expression levels of GLUT1, PKM2, and other key enzymes in aerobic glycolysis [48]. In addition, STAT3 could bind to the HK2 and LDHA gene promoter to enhance its expression [49, 50]. Here, the TCGA database analysis showed that GLUT1, HK2, and other key glycolytic enzymes were highly expressed in and negatively correlated with the survival of HCC patients, suggesting they might be used as a new generation of tumour markers. We found that STAT3 knockdown in HCC cells significantly reduced the levels of glycolysis and the maximum glycolysis capacity, and downregulated HIF‐1α as well as GLUT1, HK2, and LDHA in HCC cells, collectively indicating that STAT3 regulates glycolysis in HCC cells.

Glycolysis inhibitors, in combination with cytotoxic agents, can convert conventional cancer cell death into ICD through ERp57/calreticulin exposure on the plasma membrane [14]. Moreover, glycolysis inhibition by the glucose analogue 2‐DG in chondrocytes or acute lymphoblastic leukaemia cells considerably induces eIF2α phosphorylation‐dependent ER stress [51, 52]. We found that 2‐DG significantly inhibited HCC cell growth, which was accompanied by an increase of calreticulin surface exposure, suggesting that glycolysis is involved in the ICD of HCC cells. Glucose transport is a passive process by which extracellular glucose is transported into cells through GLUTs expressed on the cell membrane, which is the first critical step in the glycolytic cycle [53]. Among at least 13 subtypes of GLUTs identified, GLUT1, GLUT3, and GLUT4 have the highest affinity for glucose. GLUT1 has the widest intracellular distribution and a relatively high affinity for glucose, and is expressed in all normal tissues and upregulated in HCC or other tumour tissues [54]. Importantly, the GLUT1 inhibitor can block the proliferation of lung cancer, breast cancer, or other tumour cells *in vitro* and *in vivo* [55, 56]. Here, the TCGA database analysis showed that GLUT1 was positively associated with CD47 in HCC. Moreover, the GLUT1 inhibitor STF‐31 could lower glycolysis levels and promote the phosphorylation of eIF2α, inducing the ICD of HCC cells along with increased expression of calreticulin and decreased expression of CD47. In addition, STAT3 directly regulated the transcription of GLUT1 in HCC cells. Therefore, STAT3 inhibition promoted the ICD of HCC cells by inhibiting aerobic glycolysis through GLUT1.

In the subcutaneous homograft and liver orthotopic transplantation mouse models of Hepa1‐6 cells, the inhibitor of STAT3 napabucasin effectively suppressed tumour growth and prolonged the survival of HCC mice. Importantly, the tumour immune microenvironment was improved, showing increased infiltration and activation of DCs and macrophages, as well as CD8^+^ T cells with low expression of checkpoint molecules. More significantly, the antitumour immune memory induced by napabucasin enabled resistance against tumour rechallenge and recurrence *in vivo*. The present study provides evidence that STAT3 is a potential target for the treatment of HCC, inducing immunogenic cell death via blocking glycolysis and “don't eat me” molecules. Nowadays, the combination of chemical drugs with immunotherapy has achieved appreciable therapeutic effects on chronic lymphocytic leukaemia, triple‐negative breast cancer, and other types of tumours [57, 58]. Particularly, breakthroughs have been made in immunotherapy by CAR‐T and CAR‐NK cells and antibodies against immune checkpoint molecules, which have shown therapeutic effects in HCC models [59, 60, 61, 62]. It has also been confirmed that the amount of T‐cell infiltration within the tumour is positively correlated with the therapeutic effect of CAR‐T [63] and anti‐PD‐1 mAb [64]. Therefore, STAT3 inhibitors are expected to yield better results in combination with immunotherapies by improving the tumour immune microenvironment.

## 5 Conclusion

Collectively, we identified that targeting inhibition of STAT3 could induce ICD of HCC cells, evoke anti‐HCC immune responses, and immune memory *in vivo*. In addition, by highlighting the translocation of “eat me” molecules such as calreticulin to the cell surface while exposure of “don't eat me” molecule CD47 significantly decreased, STAT3 directly regulates CD47 and glycolysis via GLUT1 in HCC cells. Our findings may facilitate better understanding of targeting inhibition of STAT3 in HCC and shed new light on therapeutic strategies against HCC and other human malignancies.

## Conflict of interest

The authors declare no conflict of interest.

## Author contributions

YL conceived, performed the experiments, interpreted the data, and drafted the article. ZS, QH, and HZ were involved in study implementation, acquisition, and analysis. YL, ZP, and ZL jointly analysed the animal models. JZ initiated, designed, supervised research, and revised the article. All authors were involved in the critical review and editing of the article.

## Supporting information


**Fig. S1.** Inhibition of HCC cell growth by knockdown of STAT3 expression.

**Fig. S2.** Human PBMC‐derived hDCs.

**Fig. S3.** Doxorubicin triggers membrane translocation of ICD‐related molecules in HCC cells.

**Fig. S4.** Candidate STAT3 binding sites on human CD47 promoter region.

**Fig. S5.** High glycolysis molecule levels in HCC patients.

**Fig. S6.** Napabucasin decreases glycolysis of HCC cells *in vitro*.

**Fig. S7.** The candidate STAT3 binding sites on human GLUT1 promoter region.

**Fig. S8.** The influence of napabucasin on liver weight in liver orthotopic transplantation mouse model.

**Fig. S9.** Western blotting analysis of the effect of Napabucasin on STAT3 inactivation *in vivo*.

**Fig. S10.** The influence of napabucasin on tumour weight in subcutaneous homograft mouse model.

**Fig. S11.** The influence of napabucasin on the expression of CD95 and PD‐L1 in HCC cells.

**Fig. S12.** Napabucasin evokes antitumour immunity in HCC subcutaneous mouse model at advanced stages.

**Table S1.** Primer sequences used in qRT‐PCR.
**Table S2.** Antibodies used in western blotting.
**Table S3.** Primer sequences used in ChIP assay.
**Table S4.** Antibodies used in immunofluorescence/immunohistochemistry.
**Table S5.** Antibodies used in mouse/human studies.

## Data Availability

The data that support the findings of this study are available from the corresponding author (zhangj65@sdu.edu.cn) upon reasonable request.
