# Supplementary figures and images for: Targeted inhibition of STAT3 induces immunogenic cell death of hepatocellular carcinoma cells via glycolysis
Source: Mol Oncol. 2022 Jun 27;16(15):2861–80. doi: 10.1002/1878-0261.13263 (PMC9348600; doi:10.1002/1878-0261.13263)

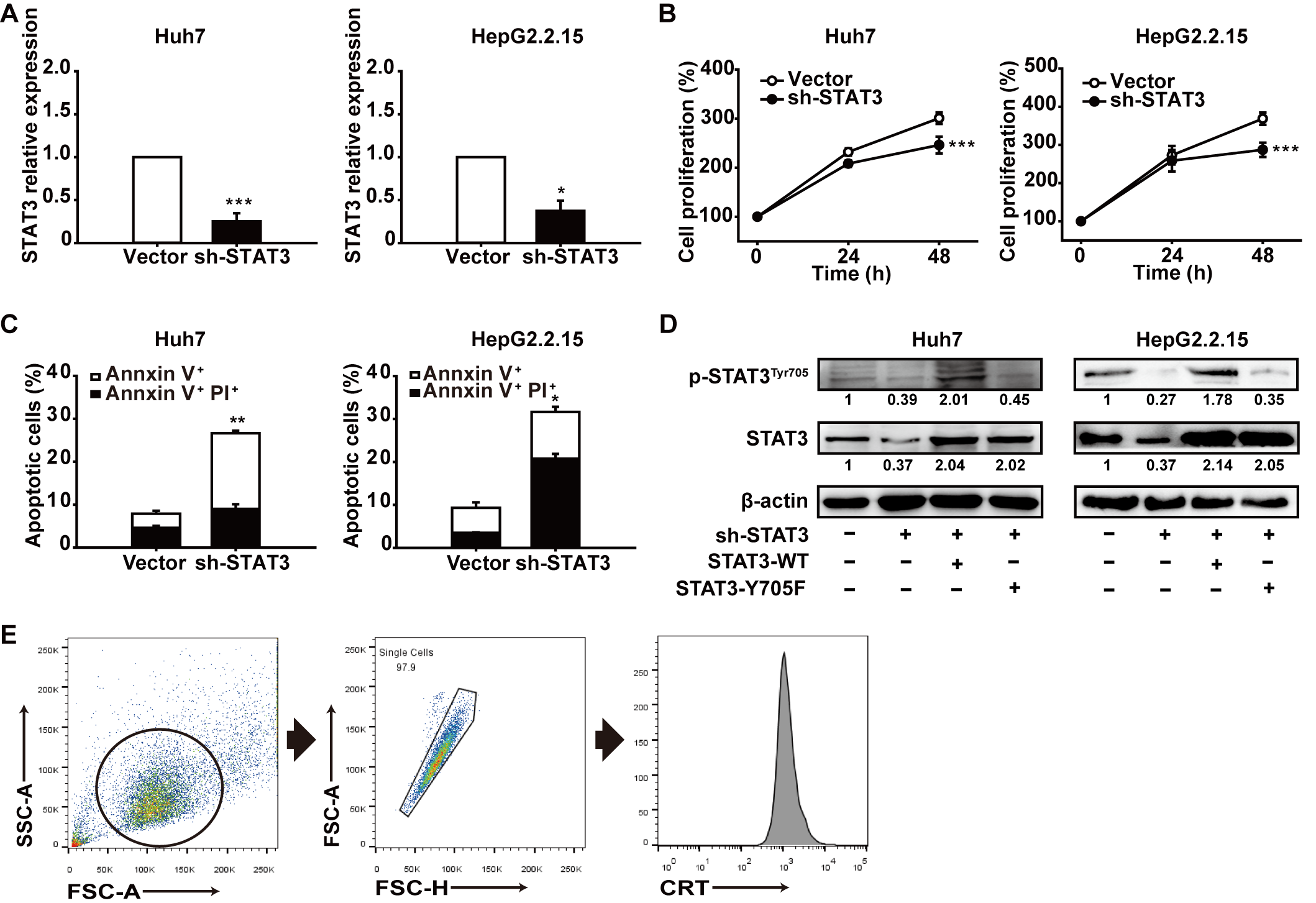

Supplement: Supplementary file 1 — Fig. S1. Inhibition of HCC cell growth by knockdown of STAT3 expression. [file MOL2-16-2861-s004.tif]

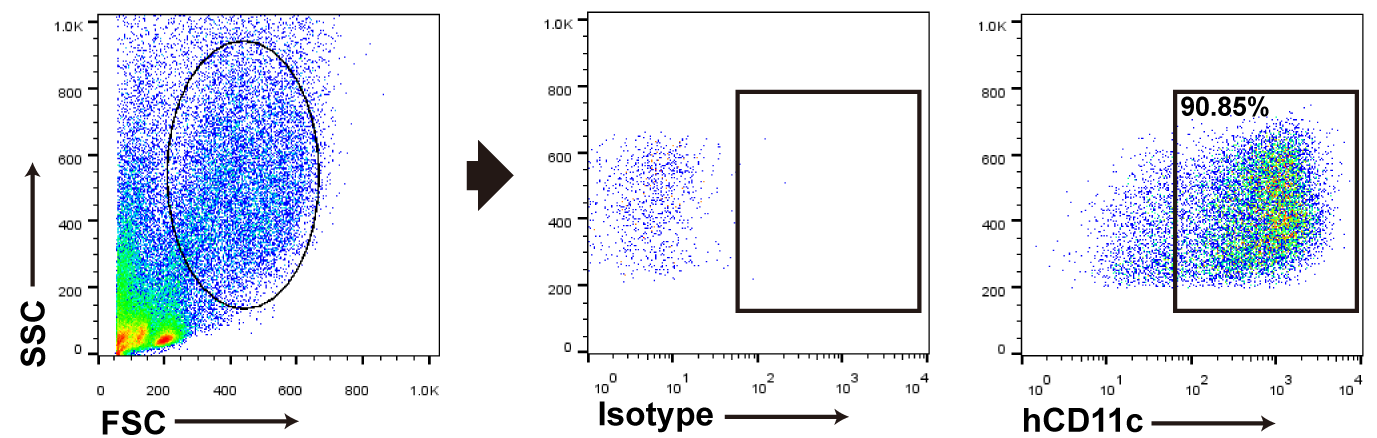

Supplement: Supplementary file 2 — Fig. S2. Human PBMC‐derived hDCs. [file MOL2-16-2861-s008.tif]

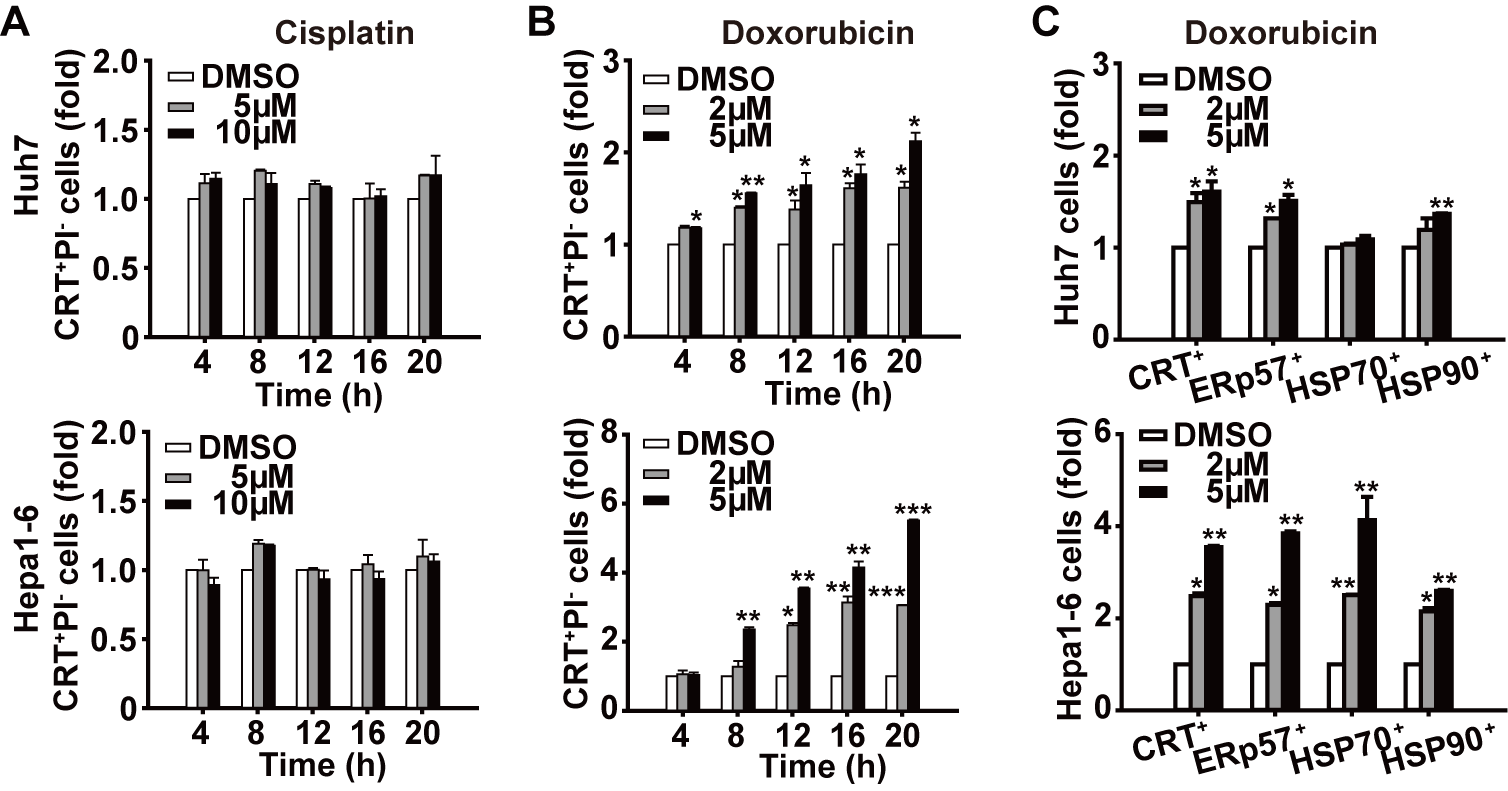

Supplement: Supplementary file 3 — Fig. S3. Doxorubicin triggers membrane translocation of ICD‐related molecules in HCC cells. [file MOL2-16-2861-s007.tif]

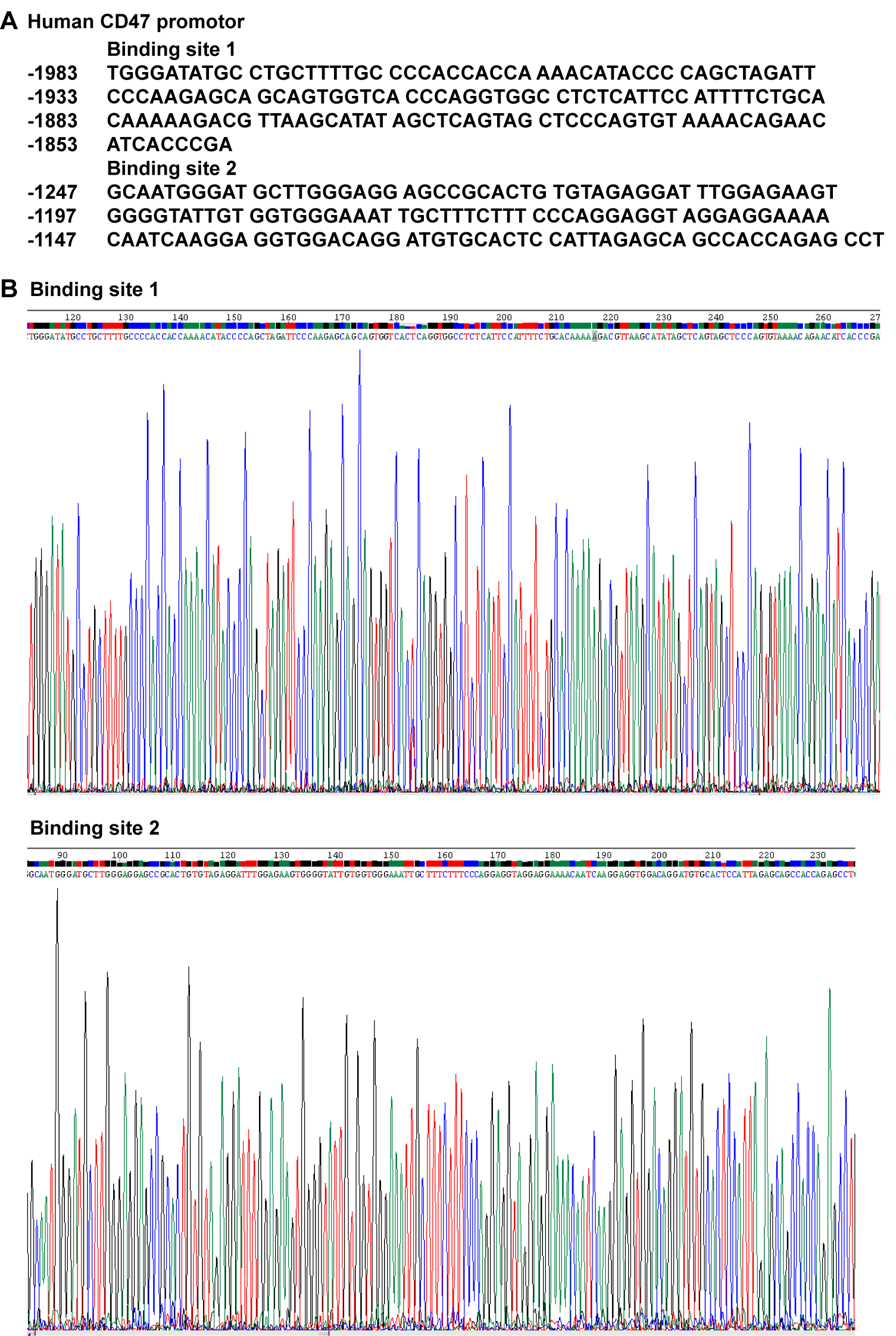

Supplement: Supplementary file 4 — Fig. S4. Candidate STAT3 binding sites on human CD47 promoter region. [file MOL2-16-2861-s012.tif]

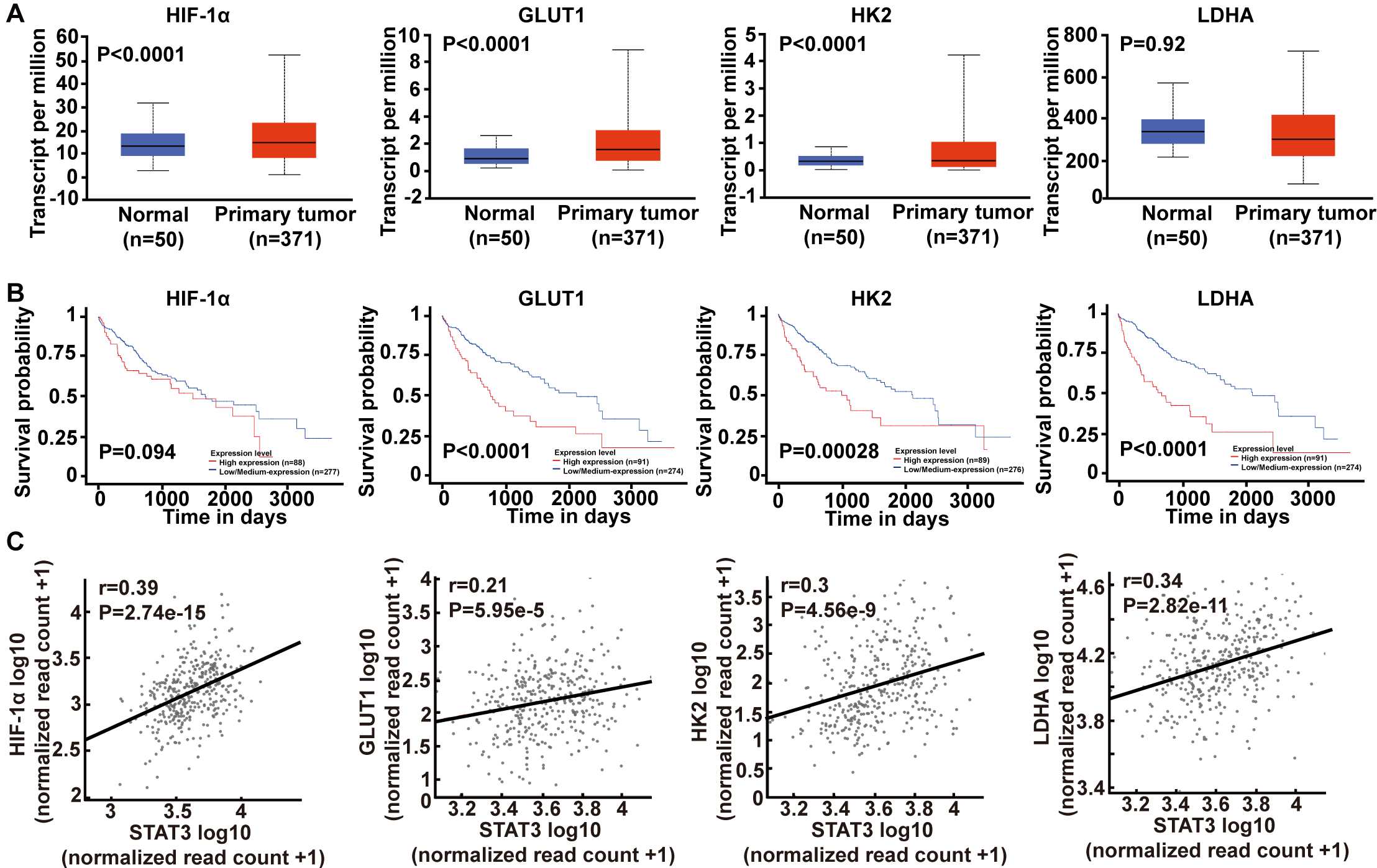

Supplement: Supplementary file 5 — Fig. S5. High glycolysis molecule levels in HCC patients. [file MOL2-16-2861-s003.tif]

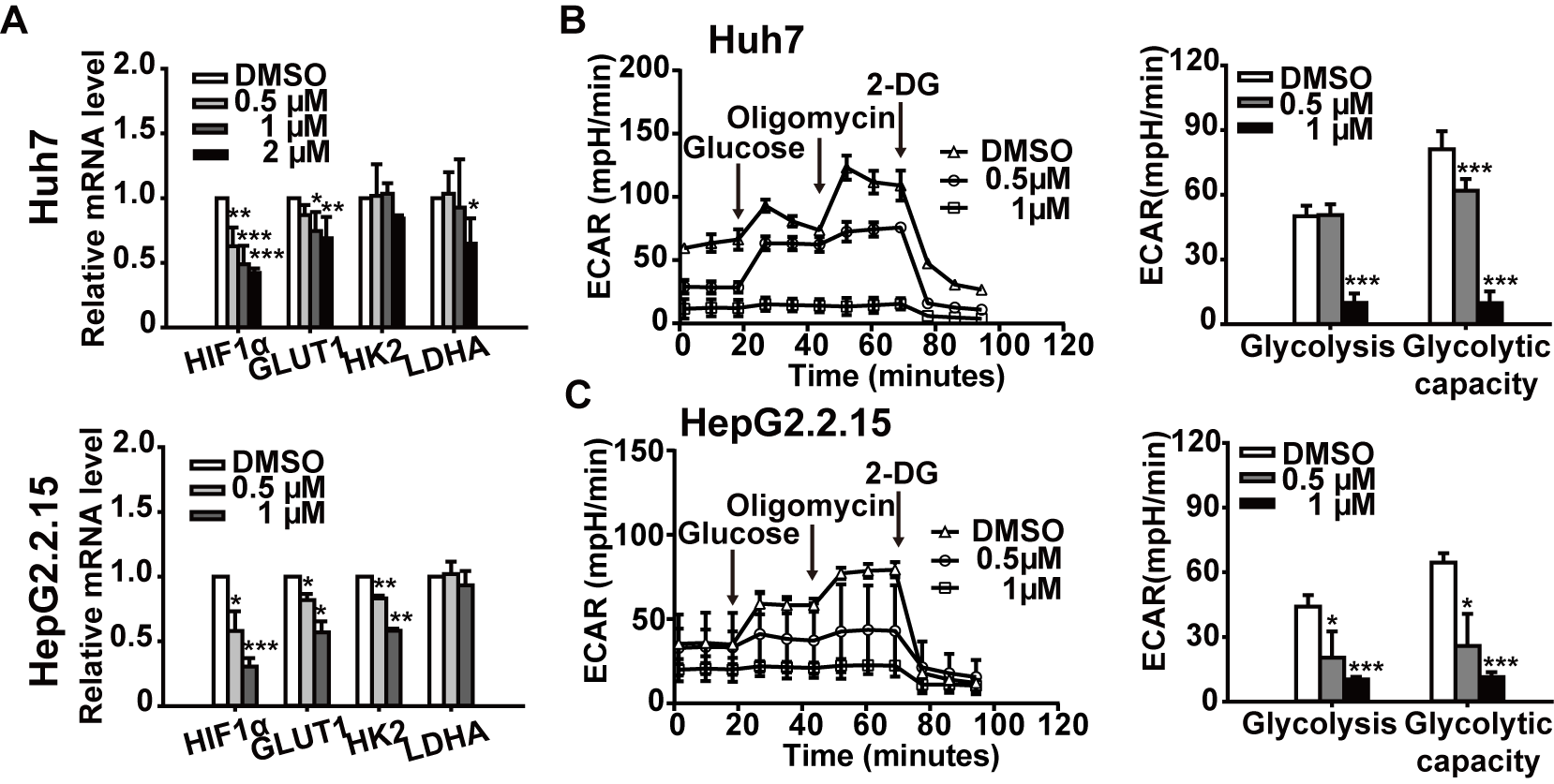

Supplement: Supplementary file 6 — Fig. S6. Napabucasin decreases glycolysis of HCC cells in vitro. [file MOL2-16-2861-s010.tif]

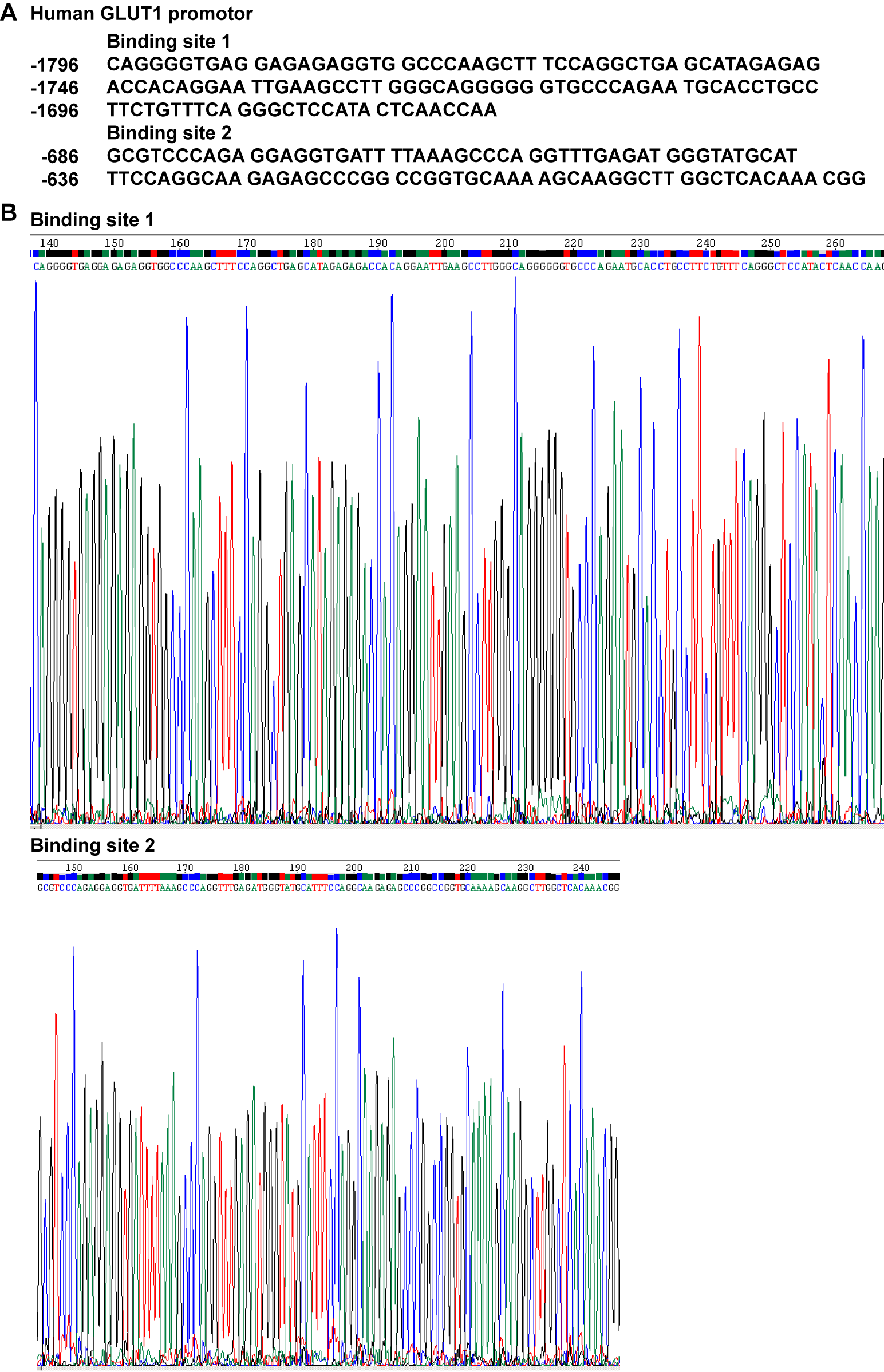

Supplement: Supplementary file 7 — Fig. S7. The candidate STAT3 binding sites on human GLUT1 promoter region. [file MOL2-16-2861-s002.tif]

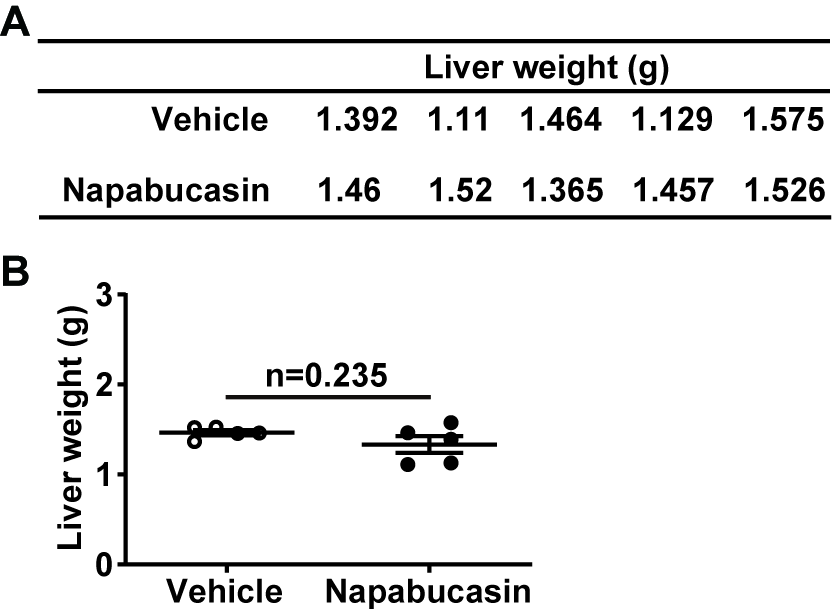

Supplement: Supplementary file 8 — Fig. S8. The influence of napabucasin on liver weight in liver orthotopic transplantation mouse model. [file MOL2-16-2861-s001.tif]

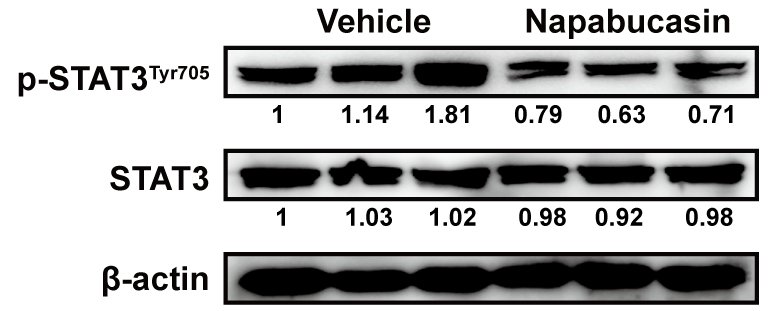

Supplement: Supplementary file 9 — Fig. S9. Western blotting analysis of the effect of Napabucasin on STAT3 inactivation in vivo. [file MOL2-16-2861-s013.tif]

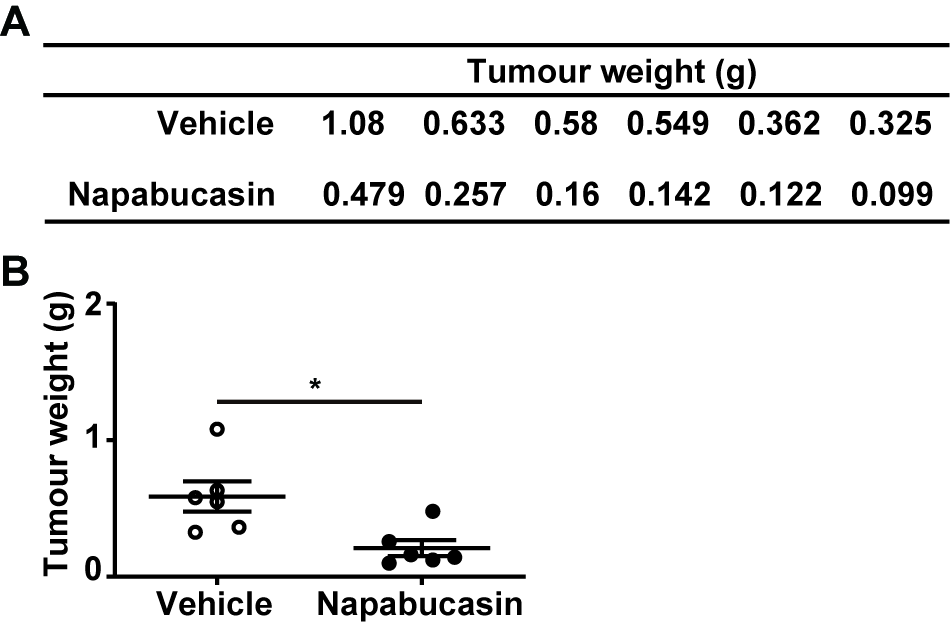

Supplement: Supplementary file 10 — Fig. S10. The influence of napabucasin on tumour weight in subcutaneous homograft mouse model. [file MOL2-16-2861-s006.tif]

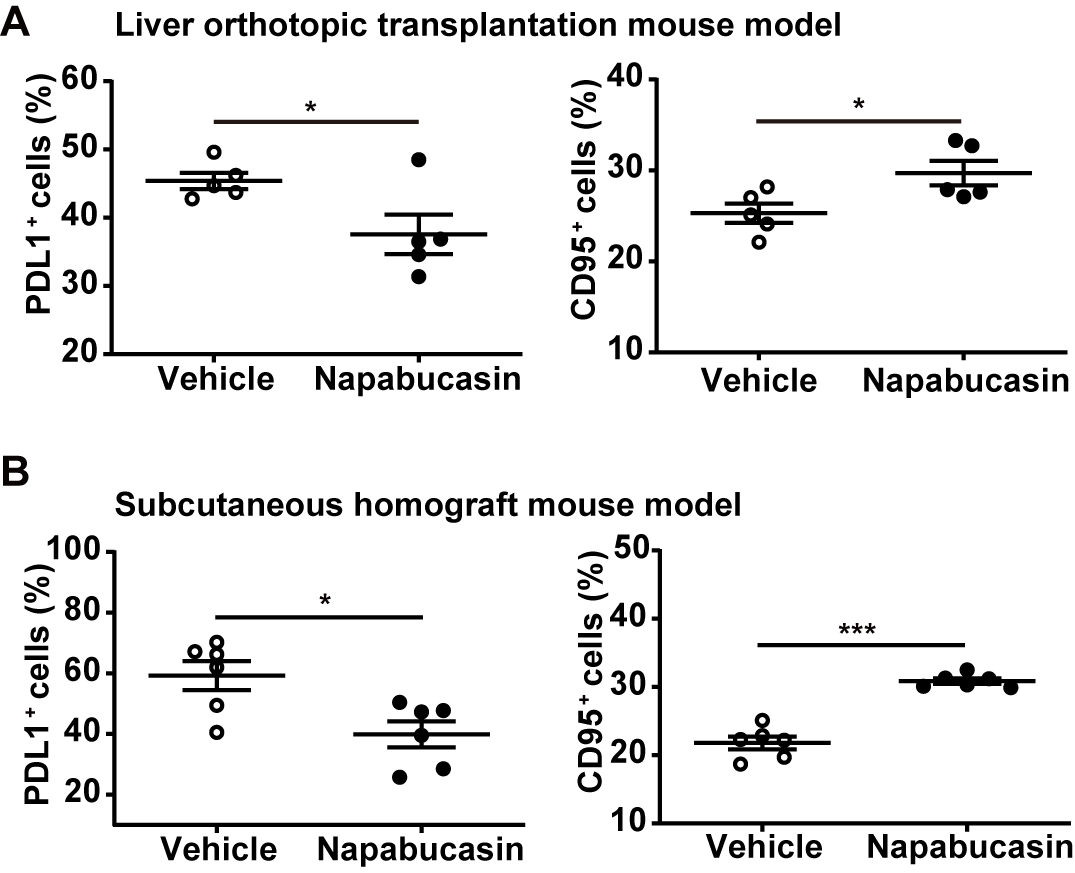

Supplement: Supplementary file 11 — Fig. S11. The influence of napabucasin on the expression of CD95 and PD‐L1 in HCC cells. [file MOL2-16-2861-s009.tif]

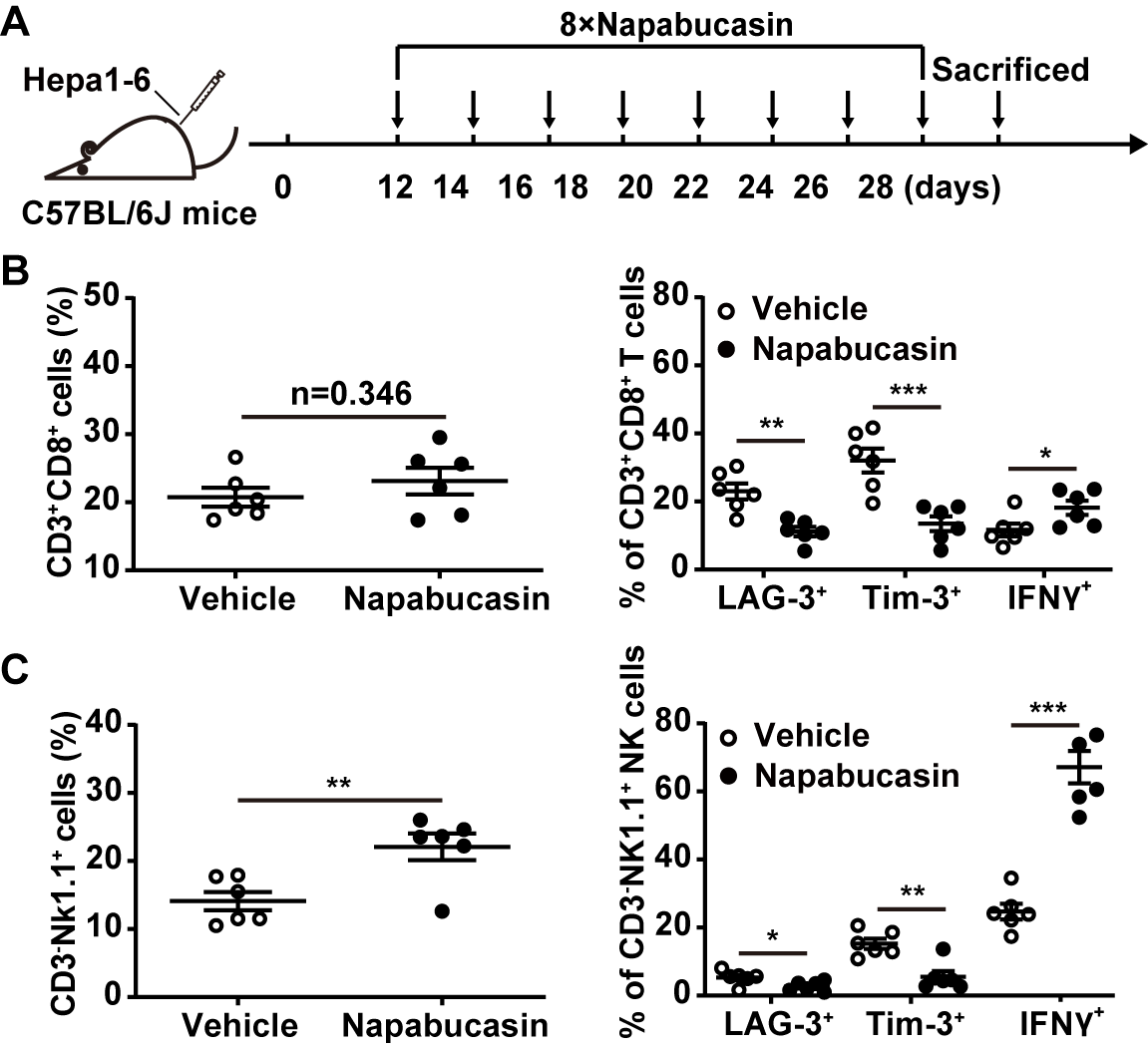

Supplement: Supplementary file 12 — Fig. S12. Napabucasin evokes antitumour immunity in HCC subcutaneous mouse model at advanced stages. [file MOL2-16-2861-s011.tif]
