# Supplementary material for: Targeted inhibition of STAT3 induces immunogenic cell death of hepatocellular carcinoma cells via glycolysis
Source: Mol Oncol. 2022 Jun 27;16(15):2861–80. doi: 10.1002/1878-0261.13263 (PMC9348600; doi:10.1002/1878-0261.13263)
Supplement: Supplementary file 13 — Table S1. Primer sequences used in qRT‐PCR. Table S2. Antibodies used in western blotting. Table S3. Primer sequences used in ChIP assay. Table S4. Antibodies used in immunofluorescence/immunohistochemistry. Table S5. Antibodies used in mouse/human studies. [file MOL2-16-2861-s005.docx]

**Targeting inhibition of STAT3 induces immunogenic cell death of hepatocellular carcinoma cells via glycolysis pathway**

Ya Li, Zhenwei Song, Qiuju Han, Huajun Zhao, Zhaoyi Pan, Zhengyang Lei, Jian Zhang

**Supplementary information:**


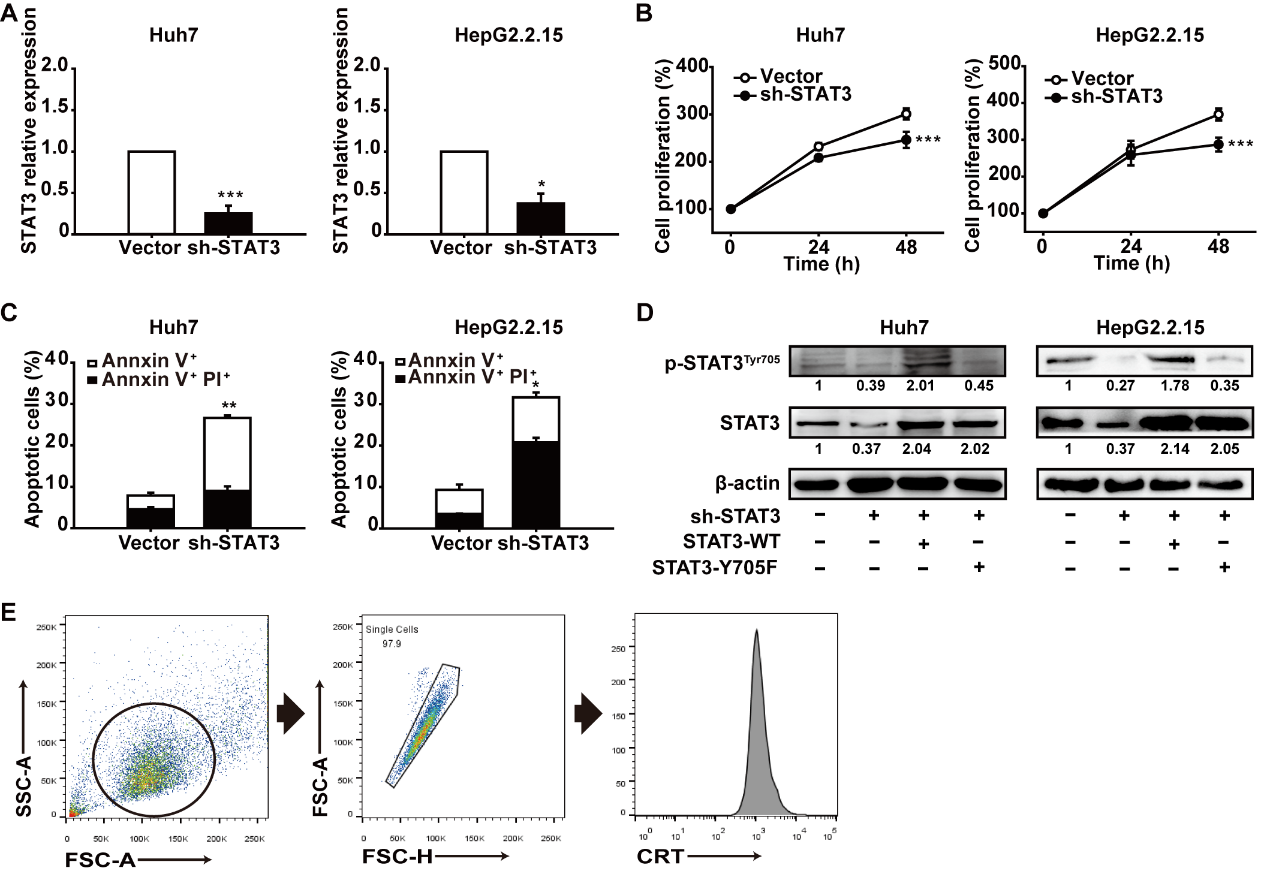


**Supplymentary Figure 1 Inhibition of HCC cell growth by knockdown of STAT3 expression.** Huh7 and HepG2.2.15 cells were infected with lentiviral STAT3-shRNA vector or empty vector for 48 h. (A) STAT3 mRNA levels in Huh7 and HepG2.2.15 cells were analysed by qRT-PCR. Data were normalised to β-actin. (B) Cell viabilities of Huh7 and HepG2.2.15 cells were analysed by CCK8 assay. (C) Huh7 and HepG2.2.15 cells were labelled with Annexin V-FITC and PI and analysed by flow cytometry. The percentage of early (white, annexin V-FITC^+^) and late (black, annexin V-FITC^+^PI^+^) apoptotic cells is shown. (D) Western blot analysis of STAT3 and p-STAT3^Tyr705^ expression in Huh7 and HepG2.2.15 cells infected with indicated lentiviral vectors. (E) The gating strategy and the expression of CRT in Huh7 cells. Data are shown as mean ± SD from three independent experiments (* P<0.05, ** P<0.01, and *** P<0.001).


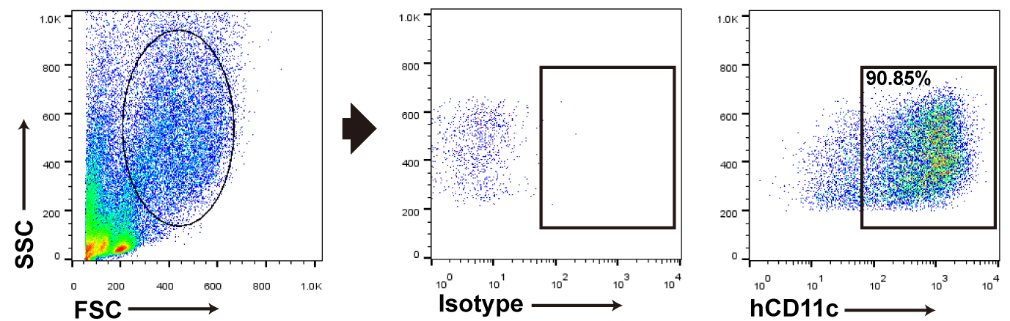


**Supplementary Figure 2** **Human PBMC-derived hDCs.** hDC precursor monocytes were purified from PBMCs by adherence, elutriation, and differentiation, and analysed by CD11c^+^ flow cytometry.


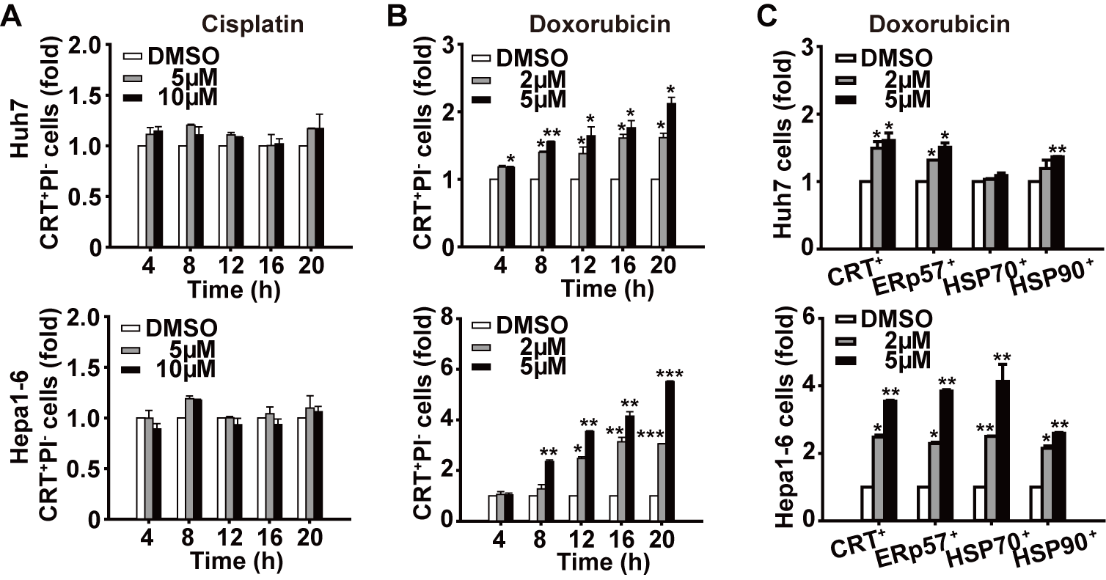


**Supplementary Figure 3 Doxorubicin triggers membrane translocation of ICD-related molecules in HCC cells.** (A&B) After treatment with cisplatin or doxorubicin at the indicated concentrations and time, Huh7 and Hepa1-6 cells were labelled with anti-calreticulin antibody and PI and analysed by flow cytometry. (C) Flow cytometry analysis of calreticulin^+^, ERp57^+^, HSP70^+^, and HSP90^+^ cells in Huh7 and Hepa1-6 cells treated with doxorubicin or DMSO for 12 h. Data are shown as mean ± SD from three independent experiments (* P<0.05, ** P<0.01, and *** P<0.001).


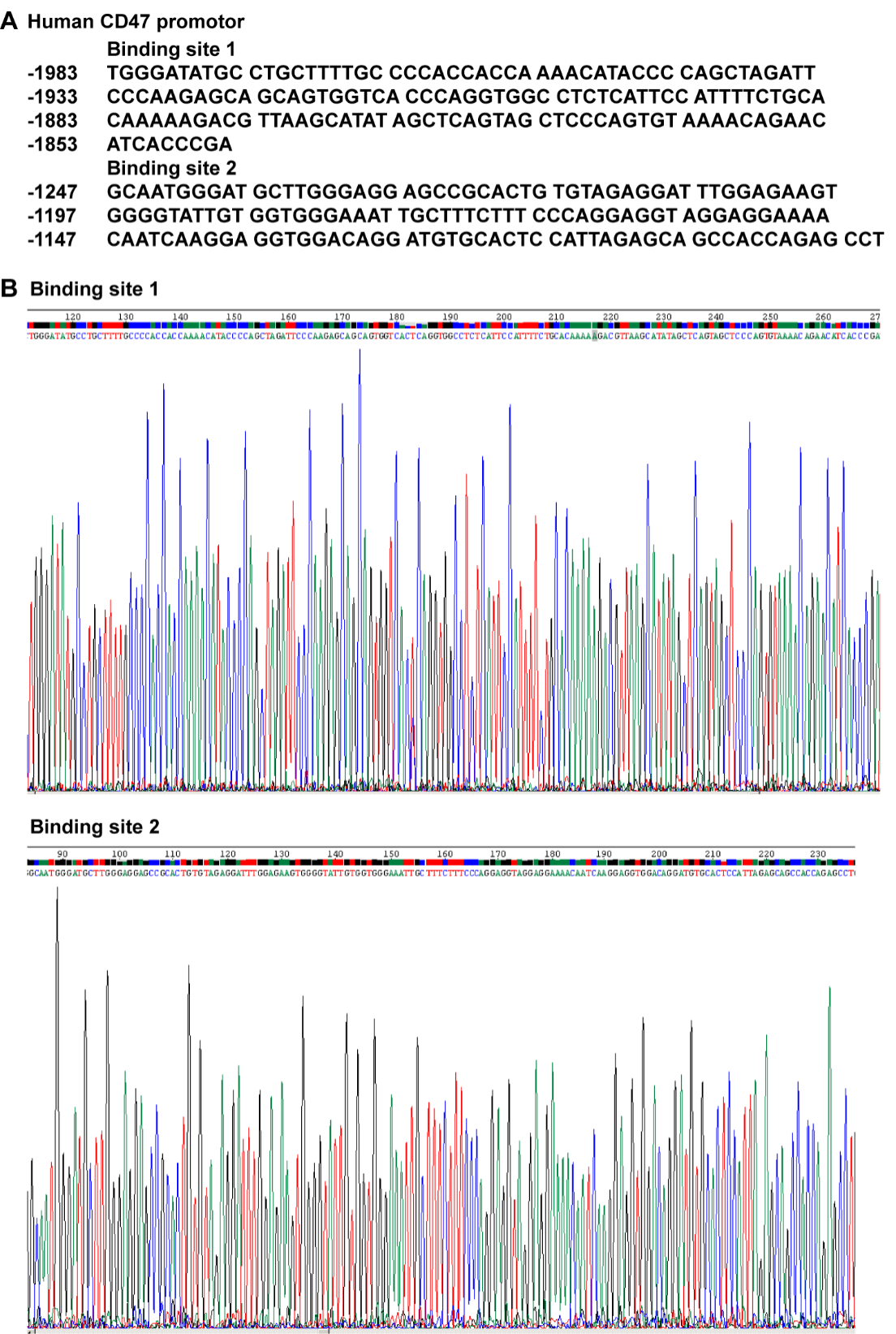


**Supplymentary Figure 4** **Candidate STAT3 binding sites on human CD47 promoter region.** (A) Base sequence of STAT3 binding sites on human CD47 promoter region. (B) Sequencing of PCR products from ChIP assay.

**
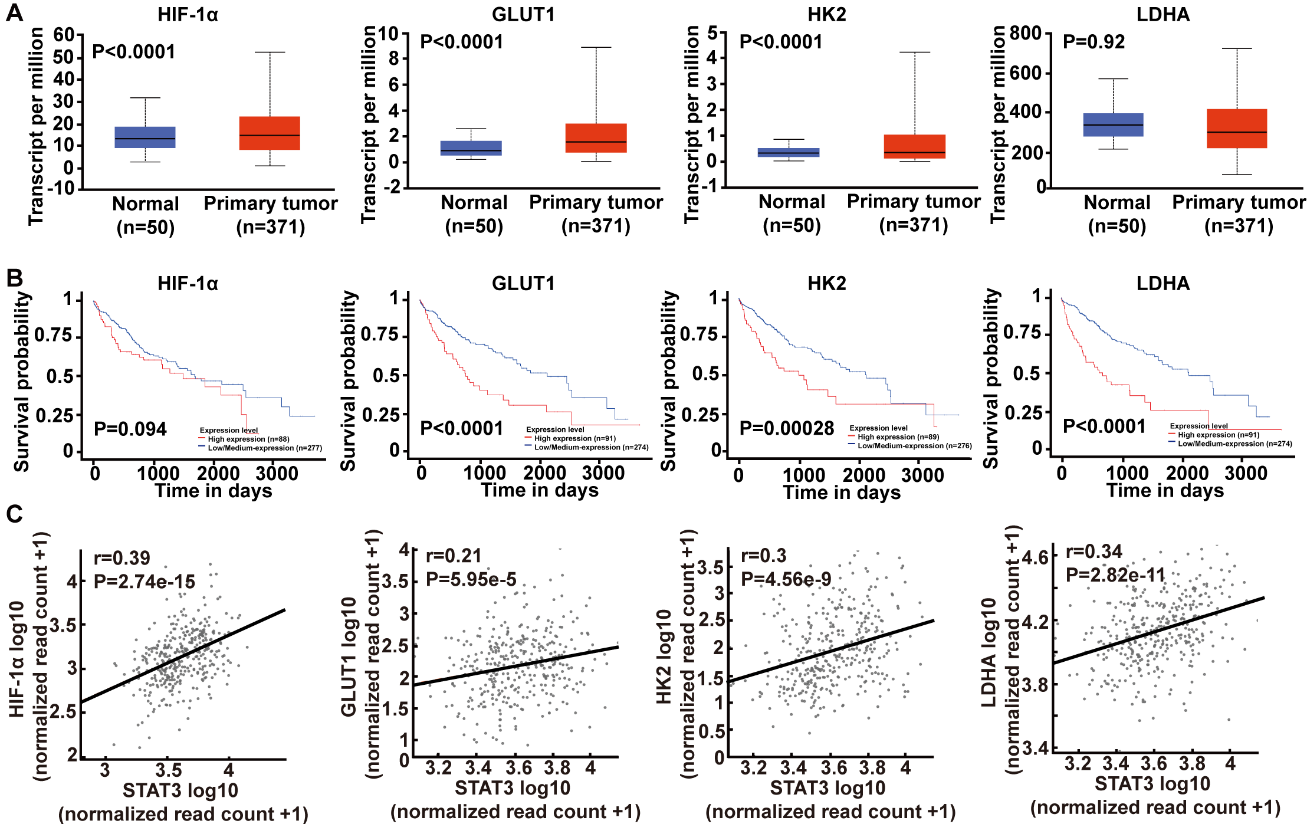
**

**Supplymentary Figure 5 High glycolysis molecule levels in HCC patients.** (A) TCGA database analysis of HIF1-α, GLUT1, HK2, and LDHA expression in HCC patients based on sample types through UALCAN database. (B) Relationship between the levels of HIF1-α, GLUT1, HK2, and LDHA in tumour tissues and survival of HCC patients in TCGA database through UALCAN database. **(**C) Validation of gene expression correlation between STAT3 and HIF1-α, GLUT1, HK2, or LDHA in HCC patients through AIPuFu database. Data from TCGA database.


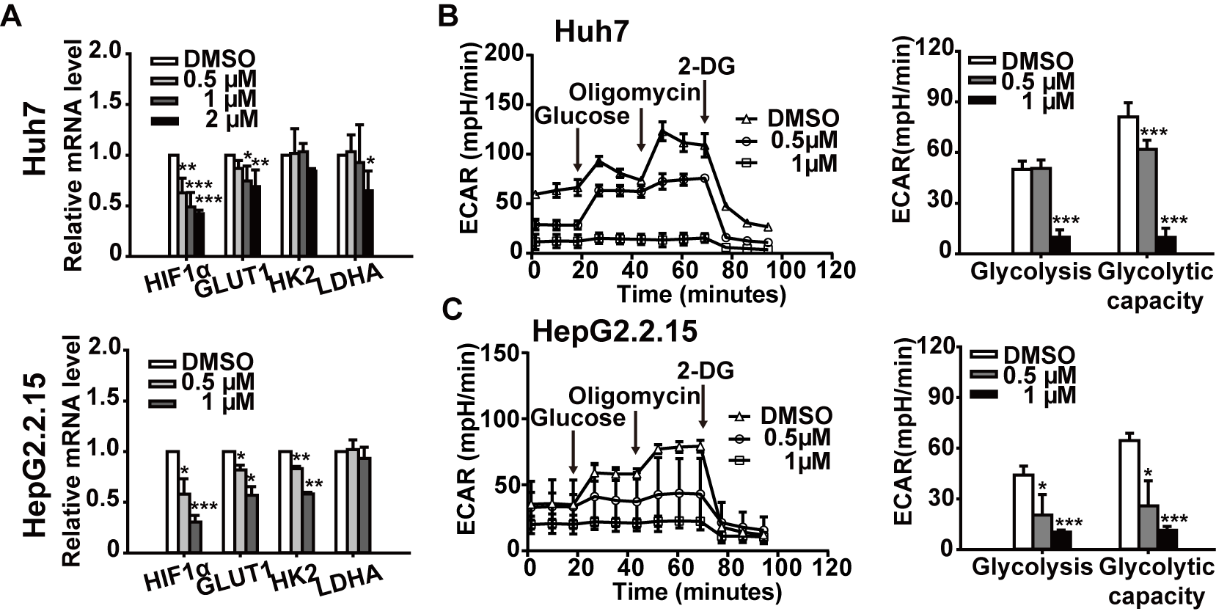


**Supplymentary Figure 6 Napabucasin decreases glycolysis of HCC cells in vitro.** (A) The mRNA levels of HIF1α, GLUT1, HK2, and LDHA in Huh7 and HepG2.2.15 cells after napabucasin or DMSO treatment were analysed by qRT-PCR. Data were normalised to β-actin and are shown as mean ± SD from three independent experiments. (B&C) The ECAR value of Huh7 and HepG2.2.15 cells after DMSO or napabucasin treatment at the indicated concentrations was automatically recorded and calculated by Seahorse XF-24 analyser (* P<0.05, ** P<0.01 and *** P<0.001).


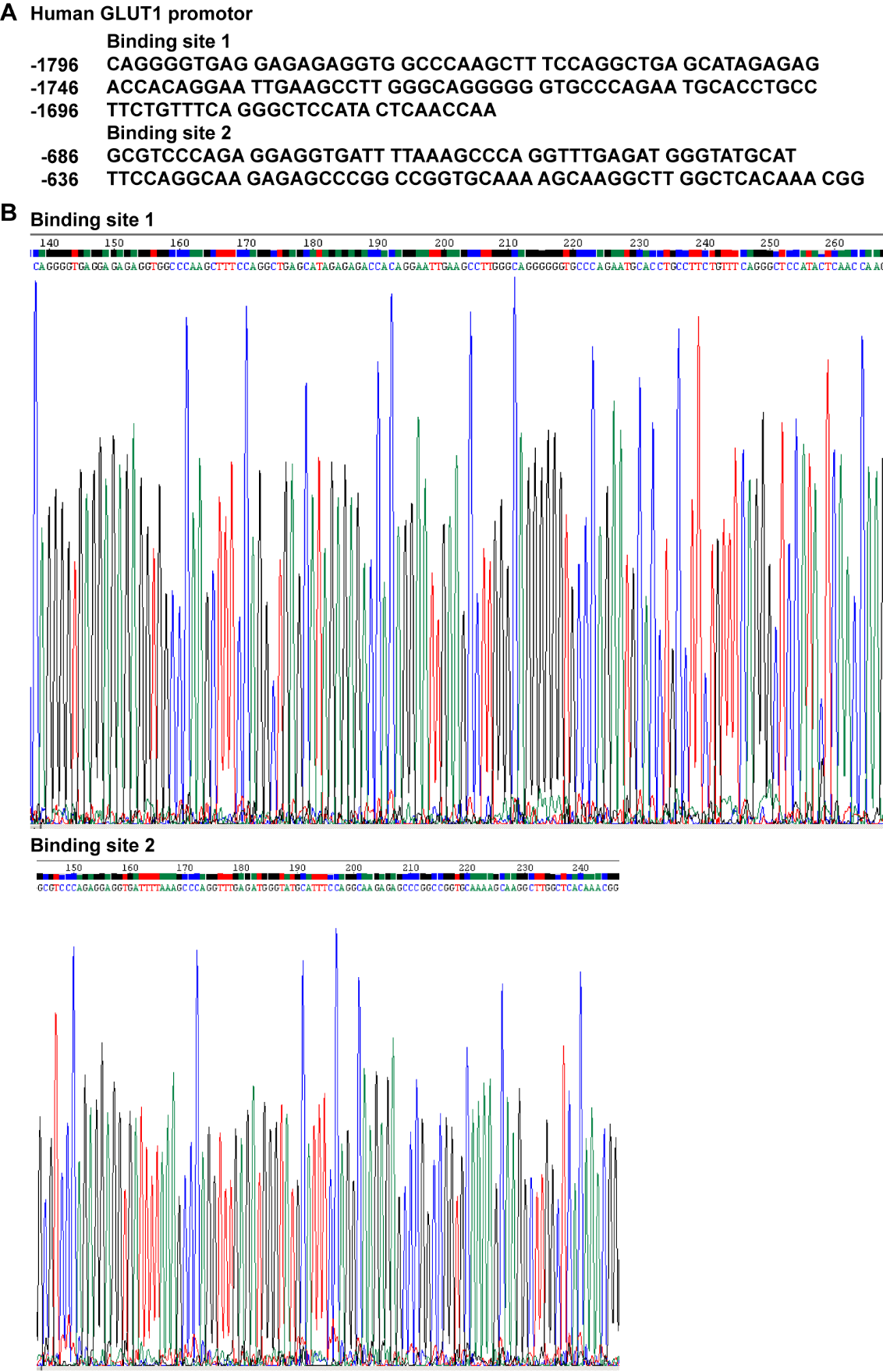


**Supplymentary Figure 7** **The candidate STAT3 binding sites on human GLUT1 promoter region.** (A) Base sequence of STAT3 binding sites on human GLUT1 promoter region. (B) Sequencing of PCR products from ChIP assay.


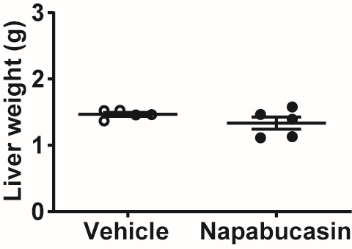


**Supplementary Figure 8 The influence of napabucasin on liver weight in liver** [**orthotopic**](javascript:;) [**transplantation**](javascript:;) **mouse model.** The weight of livers from vehicle and napabucasin treated mice in [orthotopic](javascript:;) [transplantation](javascript:;) mouse model.


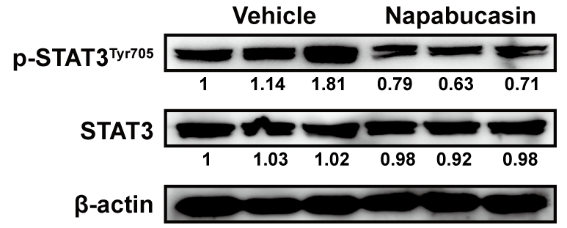


**Supplementary Figure 9 Western blotting analysis of the effect of Napabucasin on STAT3 inactivation *in vivo*.** Immunoblot showing STAT3 and p-STAT3^Tyr705^ levels in subcutaneous tumour tissues from vehicle and napabucasin treated mice.


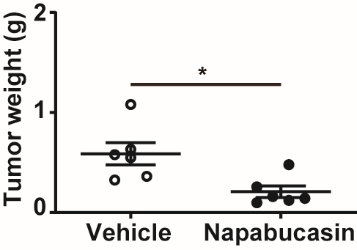


**Supplementary Figure 10 The influence of napabucasin on tumour weight in** **subcutaneous homograft mouse model.** The weight of tumours from vehicle and napabucasin treated mice in subcutaneous homograft mouse model.


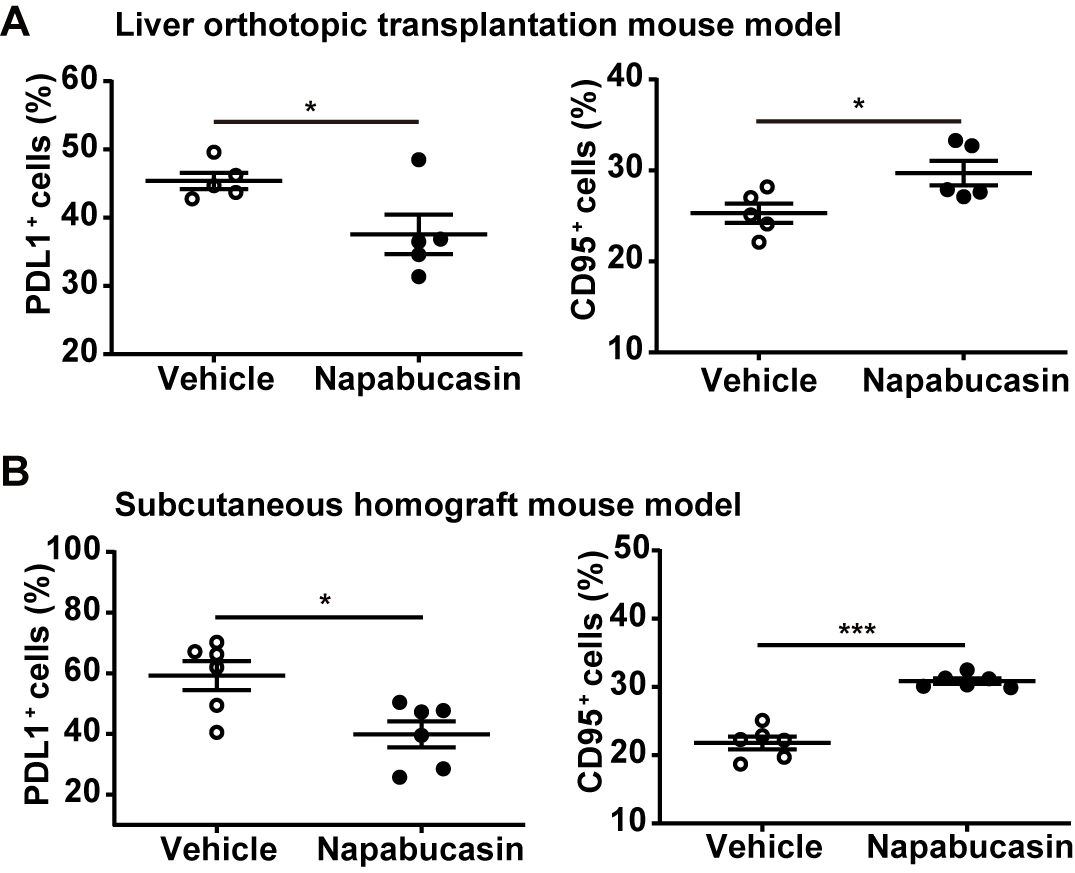


**Supplementary Figure 11 The influence of napabucasin on the expression of CD95 and PD-L1 in HCC cells.** Flow cytometry analysis of CD95^+^ and PDL1^+^ cells in liver cells from HCC orthotopic mice (n=5) (A) and tumour cells from subcutaneous HCC mice (n=6) (B). Data represent the mean ± SD of one-way ANOVA (* P<0.05, *** P<0.001).


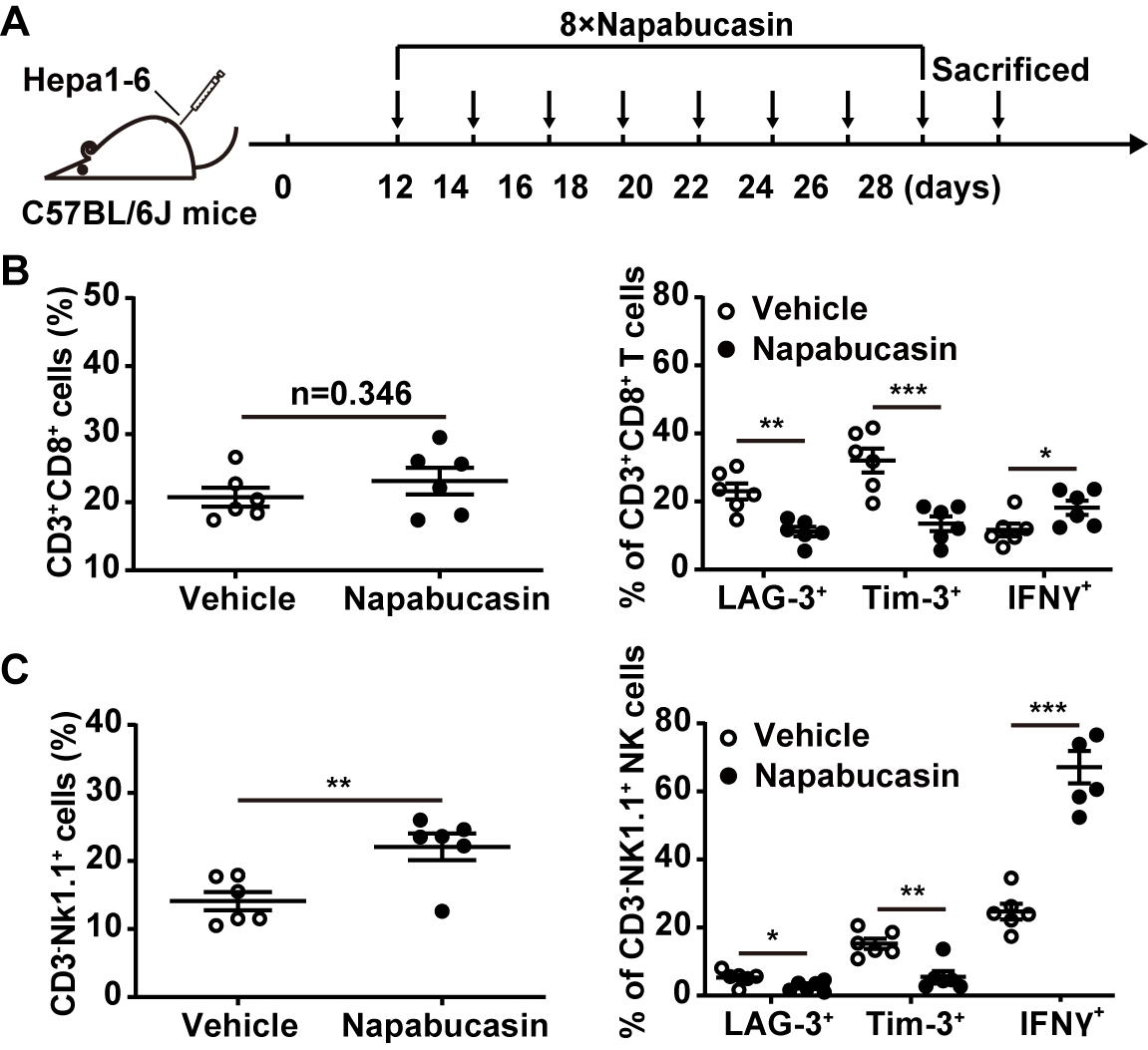


**Supplementary Figure 12 Napabucasin evokes anti-tumour immunity in HCC subcutaneous mouse model at advanced stages.** C57BL/6J mice were inoculated with 5×10^6^ Hepa1-6 cells in the left axilla. On day 12 post-inoculation, mice were intraperitoneally injected with napabucasin (20 mg/kg) every 2 days for a total of 8 injections. Solvent was used as vehicle. (A) The therapeutic schedule. (B) The proportion of CD3^+^CD8^+^ T cells and LAG-3^+^, Tim-3^+^ and IFNγ^+^ CD8^+^ T cells in tumour was analysed by flow cytometry. (C) The proportion of CD3^-^NK1.1^+^ cells and LAG-3^+^, Tim-3^+^ and IFNγ^+^ NK cells in tumour was analysed by flow cytometry. Data represent the mean ± SD of n=6 and were analysed with ANOVA (* P<0.05, ** P<0.01 and *** P<0.001).

**Table S1. Primer sequences used in qRT-PCR.**

| **Target** | **Species** | **Sequence(5′-3′)** |
| --- | --- | --- |
| β-actin | human | Forward: CATGTACGTTGCTATCCAGGC |
|  |  | Reverse: CTCCTTAATGTCACGCACGAT |
| STAT3 | human | Forward: GAGGACTGAGCATCGAGCA |
|  |  | Reverse: CATGTGATCTGACACCCTGAA |
| HIF1α | human | Forward: TTCCAGTTACGTTCCTTCGATCA |
|  |  | Reverse: TTTGAGGACTTGCGCTTTCA |
| GLUT1 | human | Forward: GGCCAAGAGTGTGCTAAAGAA |
|  |  | Reverse: ACAGCGTTGATGCCAGACAG |
| HK2 | human | Forward: TGCCACCAGACTAAACTAGACG |
|  |  | Reverse: CCCGTGCCCACAATGAGAC |
| LDHA | human | Forward: ATGGCAACTCTAAAGGATCAGC |
|  |  | Reverse: CCAACCCCAACAACTGTAATCT |
| β-actin | mouse | Forward: GGCCAACCGTGAAAAGATGA |
|  |  | Reverse: CACAGCCTGGATGGCTACGT |
| STAT3 | mouse | Forward: CAATACCATTGACCTGCCGAT |
|  |  | Reverse: GAGCGACTCAAACTGCCCT |
| HIF1α | mouse | Forward: AACACACAGCGGAGCTTTTT |
|  |  | Reverse: TTCACAAATCAGCACCAAGC |
| GLUT1 | mouse | Forward: TCTCTGTCGGCCTCTTTGTT |
|  |  | Reverse: GCAGAAGGGCAACAGGATAC |
| HK2 | mouse | Forward: ATGATCGCCTGCTTATTCACG |
|  |  | Reverse: CGCCTAGAAATCTCCAGAAGGG |
| LDHA | mouse | Forward: ACATTGTCAAGTACAGTCCACAC |
|  |  | Reverse: TTCCAATTACTCGGTTTTTGGGA |

**Table S2. Antibodies used in western blotting.**

| **Antibodies** | **Product code** | **Supplier** |
| --- | --- | --- |
| β-actin | AF5001 | Beyotime Biotechnology |
| STAT3 | D3Z2G | Cell signaling technology |
| p-STAT3^Tyr705^ | D3A7 | Cell signaling technology |
| PKR | AF2125 | Beyotime Biotechnology |
| p-PKR^Thr446^ | AF1474 | Beyotime Biotechnology |
| eIF2α | A9905 | ABclonal |
| p-eIF2α^Ser51^ | AF1237 | Beyotime Biotechnology |
| HRP-coupled goat anti-rabbit | A0208 | Beyotime Biotechnology |
| HRP-coupled goat anti-mouse | A0216 | Beyotime Biotechnology |

**Table S3. Primer sequences used in ChIP assay**

| **Target** |  | **Sequence(5′-3′)** |
| --- | --- | --- |
| CD47 | Binding site 1 | Forward: TGGGATATGCCTGCTTTT |
|  |  | Reverse: TCGGGTGATGTTCTGTTT |
|  | Binding site 2 | Forward: GCAATGGGATGCTTGGG |
|  |  | Reverse: AGGCTCTGGTGGCTGCTCTA |
| GLUT1 | Binding site 1 | Forward: CAGGGGTGAGGAGAGAGGT |
|  |  | Reverse: TTGGTTGAGTATGGAGCC |
|  | Binding site 2 | Forward: GCGTCCCAGAGGAGGTGAT |
|  |  | Reverse: CCGTTTGTGAGCCAAGCC |

**Table S4. Antibodies used in immunofluorescence/ immunohistochemistry.**

| **Antibodies** | **Product code** | **Supplier** |
| --- | --- | --- |
| Calreticulin | ab2907 | Abcam |
| ERp57 | ab10287 | Abcam |
| CD47 | ab175338 | Abcam |
| GLUT1 | ab115730 | Abcam |
| Dylight 549 Conjugated [Goat Anti-Rabbit IgG](https://www.abcam.com/goat-rabbit-igg-hl-hrp-ab205718.html)(H+L) | A23310 | Abbkine |

**Table S5. Antibodies used in mouse/human studies.**

| **Antibodies** | **Species reactivity** | **Clone** | **Supplier** |
| --- | --- | --- | --- |
| CD95 | Mouse | [SA367H8](https://www.biolegend.com/en-us/search-results?Clone=SA367H8) | BioLegend |
| PDL1 | Mouse | [MIH6](https://www.biolegend.com/en-us/search-results?Clone=MIH6) | BioLegend |
| F4/80 | Mouse | [BM8](https://www.biolegend.com/en-us/search-results?Clone=BM8) | BioLegend |
| CD80 | Mouse | [16-10A1](https://www.biolegend.com/en-us/search-results?Clone=16-10A1) | BioLegend |
| CD86 | Mouse | [GL-1](https://www.biolegend.com/en-us/search-results?Clone=GL-1) | BioLegend |
| MHCⅡ | Mouse | [M5/114.15.2](https://www.biolegend.com/en-us/search-results?Clone=M5/114.15.2) | BioLegend |
| CD4 | Mouse | [GK1.5](https://www.biolegend.com/en-us/search-results?Clone=GK1.5) | BioLegend |
| LAG-3 | Mouse | C9B7W | BioLegend |
| PD1 | Mouse | [29F.1A12](https://www.biolegend.com/en-us/search-results?Clone=29F.1A12) | BioLegend |
| Tim-3 | Mouse | RMT3-23 | BioLegend |
| CD11b | Mouse | M1/70 | eBioscience |
| CD11c | Mouse | N418 | eBioscience |
| CD3e | Mouse | 145-2C11 | eBioscience |
| IFNγ | Mouse | XMG 1.2 | eBioscience |
| CD8a | Mouse | 53-6.7 | BD Biosciences |
| CD11c | Human | 3.9 | BioLegend |
| CD80 | Human | [2D10](https://www.biolegend.com/en-us/search-results?Clone=2D10) | BioLegend |
| CD86 | Human | [BU63](https://www.biolegend.com/en-us/search-results?Clone=BU63) | BioLegend |
| CD40 | Human | [HB14](https://www.biolegend.com/en-us/search-results?Clone=HB14) | BioLegend |
| MHCⅡ | Human | [L243](https://www.biolegend.com/en-us/search-results?Clone=L243) | BioLegend |
| CD47 | Human | CC2C6 | eBioscience |
| Calreticulin | Mouse/Human | ab2907 | Abcam |
| ERp57 | Mouse/Human | ab10287 | Abcam |
| HSP70 | Mouse/Human | ab181606 | Abcam |
| HSP90 | Mouse/Human | ab203126 | Abcam |
| GLUT1 | Mouse/Human | ab115730 | Abcam |
| Rabbit IgG, polyclonal - Isotype Control | Mouse/Human | ab171870 | Abcam |
| Rabbit IgG, monoclonal - Isotype Control | Mouse/Human | ab172730 | Abcam |
| Alexa Fluor 647-conjugated secondary anti-rabbit antibody | | Jackson ImmunoResearch | |
